# Genomic sequence analysis reveals diversity of Australian *Xanthomonas* species associated with bacterial leaf spot of tomato, capsicum and chilli

**DOI:** 10.1186/s12864-019-5600-x

**Published:** 2019-04-23

**Authors:** R. Roach, R. Mann, C. G. Gambley, T. Chapman, R. G. Shivas, B. Rodoni

**Affiliations:** 1 Department of Agriculture and Fisheries, Ecosciences Precinct, Brisbane, QLD Australia; 2 Agriculture Victoria Research Division, Department of Economic Development, Jobs, Transport & Resources, AgriBio, La Trobe University, Bundoora, Victoria 3083 Australia; 3 Department of Agriculture and Fisheries, Applethorpe Research Facility, Applethorpe, QLD Australia; 4 Department of Primary Industries, Elizabeth Macarthur Agricultural Institute, Menangle, NSW Australia; 5 Centre for Crop Health, University of Southern Queensland, Toowoomba, QLD Australia

**Keywords:** Cell wall degrading enzymes, CAZymes, Secretion system, Pan genome

## Abstract

**Background:**

The genetic diversity in Australian populations of *Xanthomonas* species associated with bacterial leaf spot in tomato, capsicum and chilli were compared to worldwide bacterial populations. The aim of this study was to confirm the identities of these Australian *Xanthomonas* species and classify them in comparison to overseas isolates. Analysis of whole genome sequence allows for the investigation of bacterial population structure, pathogenicity and gene exchange, resulting in better management strategies and biosecurity.

**Results:**

Phylogenetic analysis of the core genome alignments and SNP data grouped strains in distinct clades. Patterns observed in average nucleotide identity, pan genome structure, effector and carbohydrate active enzyme profiles reflected the whole genome phylogeny and highlight taxonomic issues in *X. perforans* and *X. euvesicatoria*. Circular sequences with similarity to previously characterised plasmids were identified, and plasmids of similar sizes were isolated. Potential false positive and false negative plasmid assemblies were discussed. Effector patterns that may influence virulence on host plant species were analysed in pathogenic and non-pathogenic xanthomonads.

**Conclusions:**

The phylogeny presented here confirmed *X. vesicatoria*, *X. arboricola*, *X. euvesicatoria* and *X. perforans* and a clade of an uncharacterised *Xanthomonas* species shown to be genetically distinct from all other strains of this study. The taxonomic status of *X. perforans* and *X. euvesicatoria* as one species is discussed in relation to whole genome phylogeny and phenotypic traits. The patterns evident in enzyme and plasmid profiles indicate worldwide exchange of genetic material with the potential to introduce new virulence elements into local bacterial populations.

**Electronic supplementary material:**

The online version of this article (10.1186/s12864-019-5600-x) contains supplementary material, which is available to authorized users.

## Background

In recent years, whole genome sequences of a variety of bacterial plant pathogens have been used to investigate the phylogenetic relationships between species, as well as the genetic basis for pathogenicity and potential diagnostic target genes [1]. Next generation sequencing (NGS) and population genomics provides insight into many facets of host-pathogen interactions [2]. The wealth of information generated with NGS technology gives plant pathologists an opportunity to investigate pathogen movement, infection strategies and phenotypic trait association with the ultimate goal of providing targeted management strategies and better biosecurity. For example, genome sequence analysis of pathogenic and non-pathogenic *Xanthomonas* species on *Prunus* spp. resulted in a molecular diagnostic assay to differentiate pathogenic and non-pathogenic strains where previous tests did not [3]. Similar studies have examined the genetic diversity of *Xanthomonas* species that cause bacterial leaf spot (BLS) of tomato, capsicum and chilli worldwide [1], but not yet to Australian *Xanthomonas* strains associated with this disease.

*Xanthomonas* species reported to cause BLS in Australian tomato and pepper have been assigned to *X. euvesicatoria*, *X. perforans* and *X. vesicatoria*, with non-pathogenic strains of *X. arboricola* and *Xanthomonas* sp. also isolated [4]. A draft genome comparison of BLS-causing *X. vesicatoria*, *X. euvesicatoria*, *X. gardneri* and *X. perforans* provided the basis for many subsequent studies using genomic data. Insights into the virulence and pathogenicity of *Xanthomonas* has been provided by genomic studies that have revealed much about plasmids and secretion systems that deliver effectors and host cell wall degrading enzymes [1, 5]. Plasmid transferral via conjugation is a major mechanism of gene transfer throughout bacterial populations, accounting for rapid shifts in pathogen response to chemicals, antibiotics and host resistance genes [6–8]. Plasmids of BLS-causing *Xanthomonas* species vary in size and carry virulence and resistance genes [9, 10]. Gene cassettes and integrons are also responsible for genome diversity in *Xanthomonas* [11]. The characteristic structure and content of a number of *Xanthomonas* species has been described as an open pan genome that readily exchanges mobile elements within a population [12]. Other features of *Xanthomonas* genomes include products of bacterial secretion systems involved with host interactions such as effectors and carbohydrate active enzymes. Understanding these elements of the bacterial genome are key to understanding how genetics reflects species phylogeny and pathogenicity.

Plant defence responses to bacterial pathogens involve recognition of molecular patterns or proteins associated with bacterial secretion systems [13]. Pathogen associated molecular patterns are recognised by pattern receptors in the host that then triggers immunity. Proteins introduced by bacterial secretion systems, known as effectors may also induce immunity. Effectors of the type III secretion system (T3SS) were shown to be the main source of virulence in *X. campestris* pv. *campestris* [14], and integral to pathogenicity in *Xanthomonas* [13]. The T3SS introduces a complex of proteins to the host cell that target plant cell structures, alter the regulation of host genes or act as chaperones and delivery systems for the secreted effectors [15]. Effectors of plant pathogens are complex and diverse; some of the better studied include the TAL/ TALE (transcription activator-like) classes of effectors [16]. The Xop (*Xanthomonas* outer protein) effector classes and general effector nomenclature is described by White et al. [13], and are identified in strains of many *Xanthomonas* species. They note that the complex interactions between secreted proteins and host cells will likely be expanded and refined with additional genomic data. The need to understand the impact of effectors is demonstrated by the *X. perforans* host range expansion partially correlated with the loss of the effector AvrBsT [17]. Interestingly, AvrBsT has been described as a fitness factor [18], demonstrating that effectors may influence disease severity as well as host range. Other effectors have been linked to pathogenic function, such as AvrHah1 inducing a water soaking effect common in many bacterial diseases [19] by upregulating the intake of water into cells. Even as more genomes are sequenced every year, there is still much to be investigated about effector function [20].

In addition to the T3SS, the type II secretion system (T2SS) has also been described as important for pathogenicity in *Xanthomonas* species [21]. The type II secretion system is a common feature of many plant and animal pathogens as well as non-pathogenic species, involved in a range of infection and colonisation processes [22]. The T2SS is typically associated with the secretion of carbohydrate active enzymes (CAZymes), families of enzymes involved in carbohydrate processing pathways. Carbohydrate degradation has traditionally been used as a diagnostic trait in bacteriology [23], and have also been discussed in structural biology as therapeutic targets [24]. Determining which CAZyme families are present in bacterial strains may indicate substrate preference and pathogenicity. As they are currently classified, CAZymes are described by protein sequence as numbered families of six classes; glycoside hydrolases (GH), glycosyl transferases (GT), polysaccharide lyases (PL), carbohydrate esterases (CE), carbohydrate binding modules (CBM), and auxiliary activity families (AA) [25, 26]. The variety of secreted proteins in *Xanthomonas* and their impact on pathogenicity has been reviewed previously [1, 27], highlighting the potential for effector and CAZyme profiles to infer pathogenicity and bacterial growth strategies.

The analyses of genome structure and secretion system products contribute to the understanding of bacterial relatedness and function. By comparing genomes of Australian BLS-associated *Xanthomonas* strains we aim to improve our understanding of the taxonomic status of these species as well as incorporating Australian BLS-causing strains into the global understanding of this pathogen complex. These analyses will provide a foundation for further identification of targets for resistance breeding or future population genetics studies.

## Results

### Taxonomy and pathogenicity

Genome statistics of all 50 Australian *Xanthomonas* draft genomes are reported in Table 1. All draft genomes were approximately 5 Mbp in length, ranging from 4,806,110 bp to 5,379,097 bp and had a GC content ranging from 64.02 to 66.14% (average of 64.74%) which is consistent with reference genomes of the sequenced species [10, 27].

**Table 1:** Draft genome statistics of 50 Australian *Xanthomonas* strains and reported genome statistics of public assemblies from Genbank

Strain	Genbank ID ^b^	Species ^c^	Host	Region	Collection date	Contig number	Mb	CDS ^f^	N50	GC%	Reference
BRIP^a^ 62410	QFAP01	xa	Tomato	Stanthorpe	2015	36	4.9	4077	224,446	65.76	this study
BRIP 62412	QFAO01	xa	Tomato	Stanthorpe	2015	53	5.12	4295	403,341	65.65	this study
BRIP 62414	QFAN01	xa	Tomato	Stanthorpe	2015	45	5.14	4304	307,902	65.63	this study
BRIP 62416	QFAM01	xa	Tomato	Stanthorpe	2015	63	5.17	4266	455,337	65.66	this study
BRIP 62432	QFAQ01	xa	Tomato	Tenterfield	2015	49	4.95	4060	227,845	65.61	this study
NCPPB 1630	JPHE01	xa pv ce ^d^	Banana	New Zealand	1960	121	4.98	4062	80,994	65.5	Harrison et al. 2014 (Unpublished)
NCPPB 1832	JPHC01	xa pv ce	Banana	New Zealand	1960	75	4.91	4044	172,772	65.6	Harrison et al. 2016
NCPPB 1447	AJTL01	xa pv j ^d^	na ^e^	na	na	371	5.02	4257	26,883	65.4	Noh et al. (Unpublished)
NCCB 100457	APMC02	xa pv co ^d^	Hazel	USA	2010	281	5.23	4346	35,479	65.5	Ibarra Caballero et al. 2013
xa3004	AZQY01	xa	Barley	Russia	1998	132	4.76	3936	61,665	66	Ignatov et al. 2015
IVIA 2626.1	LJGN01	xa pv p ^d^	Plum (*Prunus salicina*)	Spain	2009	214	5.03	4268	47,650	65.4	Garita-Cambronero et al. 2016
CITA 44	LJGM01	xa	Cherry (*Prunus mahaleb*)	Spain	2002	71	4.76	3951	120,981	65.8	Garita-Cambronero et al. 2016
J303	LSGZ01	xa pv j	Walnut	Chile	2011	161	5.07	4227	53,673	65.5	Santander et al. (Unpublished)
CITA 124	LXKK01	xa	Peach (*Prunus persica*)	Spain	2012	128	4.75	4041	60,818	65.8	Garita-Cambronero et al. (Unpublished)
CITA 14	LXIB01	xa	Peach (Prunus persica)	Spain	2008	72	4.86	4001	100,946	65.6	Garita-Cambronero et al. (Unpublished)
CFBP 2528	JZEF01	xa pv j	Walnut	New Zealand	1956	19	5.08	4264	431,859	65.5	Cesbron et al. (Unpublished)
CFBP 7634	JZEH01	xa	Walnut	France	2002	5	4.93	4102	3,063,886	65.6	Cesbron et al. (Unpublished)
CFBP 7179	JZEG01	xa pv j	Walnut	France	2002	23	5.16	4358	479,113	65.4	Cesbron et al. (Unpublished)
CFBP 7651	JZEI01	xa	Walnut	France	2008	8	5.03	4188	2,811,770	65.5	Cesbron et al. (Unpublished)
CFBP 3894	LOMI01	xa pv p	Plum (Prunus salicina)	New Zealand	1953	78	5.06	4204	162,135	65.4	Lopez-Soriano et al. (Unpublished)
MAFF301420	BAVC01	xa pv p	na	na	na	396	5.002	4485	21,373	65.3	Fujikawa et al. (Unpublished)
MAFF311562	BAVB01	xa pv p	na	na	na	432	5.08	4630	23,374	65.3	Fujikawa et al. (Unpublished)
MAFF301427	BAVD01	xa pv p	na	na	na	356	4.9	4444	27,529	65.4	Fujikawa et al. (Unpublished)
Xap33	JHUQ01	xa pv p	Almond	Spain	2009	474	5.1	4388	34,438	65.4	Garita-Cambronero et al. 2014
BRIP 63464	QEZX01	xe	Capsicum	Bundaberg	2015	140	5.34	4561	194,822	64.53	this study
DAR 82542	QEYY01	xe	Capsicum	Bundaberg	1981	426	5.12	4482	30,590	64.66	this study
BRIP 62441	QFAC01	xe	Chilli	Bundaberg	2015	135	5.34	4565	159,122	64.52	this study
BRIP 62555	QFAB01	xe	Chilli	Bundaberg	2015	139	5.34	4570	164,574	64.53	this study
BRIP 62757	QFAA01	xe	Chilli	Bundaberg	2015	149	5.33	4558	167,015	64.53	this study
BRIP 62858	QEZZ01	xe	Chilli	Bundaberg	2015	144	5.34	4554	146,014	64.53	this study
BRIP 62959	QEZY01	xe	Chilli	Bundaberg	2015	129	5.34	4560	169,175	64.53	this study
BRIP 38997	QEZV01	xe	Chilli	Bundaberg	1986	112	5.3	4513	163,565	64.55	this study
BRIP 39016	QEZW01	xe	Apple of Peru (*Nicandra physalodes*)	Christmas creek	1973	96	5.06	4290	171,827	64.6	this study
DAR 34895	QEYX01	xe	Capsicum	Dural	1981	69	5.23	4455	176,952	64.62	this study
BRIP 62390	QFAL01	xe	Capsicum	Gatton	2014	137	5.34	4550	153,551	64.53	this study
BRIP 62391	QFAK01	xe	Capsicum	Gatton	2014	131	5.34	4559	113,982	64.53	this study
BRIP 62392	QFAJ01	xe	Capsicum	Gatton	2014	183	5.34	4562	169,141	64.53	this study
BRIP 62395	QFAI01	xe	Capsicum	Gatton	2014	169	5.35	4574	179,351	64.53	this study
BRIP 62396	QFAH01	xe	Chilli	Gatton	2014	161	5.38	4604	102,920	64.5	this study
BRIP 62400	QFAG01	xe	Chilli	Gatton	2014	133	5.34	4548	194,822	64.53	this study
BRIP 62403	QFAF01	xe	Chilli	Gatton	2014	144	5.34	4557	135,453	64.53	this study
BRIP 62438	QFAD01	xe	Capsicum	Hawkesbury Heights	2015	99	5.12	4310	161,933	64.71	this study
BRIP 62425	QFAE01	xe	Capsicum	Stanthorpe	2015	163	5.34	4576	164,091	64.53	this study
DAR 26930	QEYW01	xe	Tomato	Liverpool	1976	101	5.05	4201	103,617	64.9	this study
66b	JSZG01	xe	Capsicum (*C. annuum* cv. Kambi)	Bulgaria	2012	384	5.31	4550	28,793	64.6	Vancheva et al. 2015
83 M	JSZH01	xe	Capsicum (C. annuum cv. Kurtovska kapija)	Macedonia	2013	317	5.1	4330	31,385	64.7	Vancheva et al. 2015
LMG 27970	JPYC01	xe	pepper	Belgium	1947	1491	5.04	4380	9186	64.7	Constantin et al. 2015
Xe329	JZLQ01	xe	Pepper	USA	1997	203	5.09	4242	210,541	64.7	Schwartz et al. 2015
Xe206	JZLN01	xe	Pepper	USA	1995	133	5.44	4648	133,864	64.5	Schwartz et al. 2015
Xe515	JZLV01	xe	Pepper	USA	1999	118	5.32	4525	163,350	64.5	Schwartz et al. 2015
Xe526	JZLW01	xe	Pepper	USA	2000	178	5.39	4598	176,627	64.5	Schwartz et al. 2015
Xe586	JZLX01	xe	Pepper	USA	2003	120	5.31	4522	140,453	64.6	Schwartz et al. 2015
Xe679	JZLZ01	xe	Pepper	USA	2008	109	5.14	4324	144,310	64.7	Schwartz et al. 2015
Xe681	JZMA01	xe	Pepper	USA	2009	104	5.16	4360	210,555	64.7	Schwartz et al. 2015
Xe678	JZLY01	xe	Pepper	USA	2008	202	5.42	4643	132,312	64.4	Schwartz et al. 2015
Xe259	JZLO01	xe	Pepper	USA	1995	139	5.25	4423	187,535	64.6	Schwartz et al. 2015
Xe199	JZLM01	xe	Pepper	USA	1994	110	5.27	4470	174,842	64.6	Schwartz et al. 2015
Xe315	JZLP01	xe	Pepper	USA	1996	629	5.51	4638	164,442	64.3	Schwartz et al. 2015
Xe354	JZLR01	xe	Pepper	USA	1998	287	5.43	4629	176,627	64.4	Schwartz et al. 2015
Xe376	JZLS01	xe	Pepper	USA	1995	128	5.3	4500	176,635	64.5	Schwartz et al. 2015
Xe455	JZLT01	xe	Pepper	USA	1998	137	5.33	4549	148,783	64.5	Schwartz et al. 2015
Xe490	JZLU01	xe	Pepper	USA	1999	132	5.37	4587	187,439	64.5	Schwartz et al. 2015
Xe683	JZRP01	xe	Pepper	USA	2010	108	5.32	4544	163,371	64.5	Schwartz et al. (Unpublished)
Xe684	JZRQ01	xe	Pepper	USA	2010	157	5.38	4594	148,783	64.5	Schwartz et al. (Unpublished)
Xe685	JZRR01	xe	Pepper	USA	2010	131	5.32	4536	148,770	64.5	Schwartz et al. (Unpublished)
F4–2	JZRU01	xe	Pepper	USA	2004	123	5.35	4591	159,113	64.5	Schwartz et al. (Unpublished)
H3–2	JZRW01	xe	Pepper	USA	2004	108	5.32	4531	163,306	64.5	Schwartz et al. (Unpublished)
Xe689	JZRS01	xe	Pepper	USA	2011	143	5.37	4588	163,336	64.5	Schwartz et al. (Unpublished)
Xe695	JZRT01	xe	Pepper	USA	2012	110	5.36	4588	163,310	64.5	Schwartz et al. (Unpublished)
G4–1	JZRV01	xe	Pepper	USA	2004	112	5.35	4586	163,306	64.5	Schwartz et al. (Unpublished)
L3–2	JZRX01	xe	Pepper	USA	2004	152	5.36	4584	159,114	64.5	Schwartz et al. (Unpublished)
Xe181	JZRY01	xe	Pepper	USA	1994	107	5.31	4518	163,975	64.5	Schwartz et al. (Unpublished)
ATCC 19865	AEQX01	xg	Tomato	Yugoslavia	1961	552	5.53	4786	22,686	63.7	Potnis et al. 2011
SM406–11	JZJW01	xg	Tomato	USA	2011	461	5.26	4508	18,995	63.5	Schwartz et al. 2015
SM234–10	JZJV01	xg	Tomato	USA	2010	250	5.33	4521	42,583	63.5	Schwartz et al. 2015
SM413–11	JZJX01	xg	Tomato	USA	2011	313	5.31	4527	29,049	63.5	Schwartz et al. 2015
SM605–11	JZJY01	xg	Tomato	USA	2011	242	5.34	4517	45,830	63.5	Schwartz et al. 2015
SM194–10	JZJR01	xg	Tomato	USA	2010	309	5.39	4550	41,678	63.5	Schwartz et al. 2015
SM177–10	JZJS01	xg	Tomato	USA	2010	245	5.31	4509	41,491	63.5	Schwartz et al. 2015
SM182–10	JZJT01	xg	Tomato	USA	2010	251	5.3	4505	36,743	63.5	Schwartz et al. 2015
SM220–10	JZJU01	xg	Tomato	USA	2010	260	5.29	4506	38,544	63.5	Schwartz et al. 2015
SM795–12	JZKA01	xg	Tomato	USA	2012	293	5.13	4325	37,712	63.6	Schwartz et al. 2015
SM775–12	JZJZ01	xg	Tomato	USA	2012	241	5.24	4420	45,190	63.6	Schwartz et al. 2015
BRIP 62383	QEZU01	xp	Tomato	Bowen	2012	61	5.27	4462	302,799	64.68	this study
BRIP 62384	QEZT01	xp	Tomato	Bowen	2012	77	5.25	4450	312,509	64.66	this study
BRIP 62398	QEZP01	xp	Tomato	Bowen	2014	57	5.23	4410	187,438	64.67	this study
BRIP 62404	QEZO01	xp	Tomato	Bowen	2014	109	5.25	4446	140,260	64.66	this study
BRIP 62397	QEZQ01	xp	Tomato	Brisbane	2014	45	4.97	4178	288,612	64.99	this study
BRIP 62386	QEZS01	xp	Tomato	Bundaberg	2012	80	5.29	4484	187,311	64.67	this study
BRIP 62405	QEZN01	xp	Tomato	Bundaberg	2014	77	5.28	4478	293,980	64.67	this study
BRIP 63262	QEZM01	xp	Tomato	Bundaberg	2015	100	5.33	4545	372,915	64.65	this study
BRIP 63565	QEZL01	xp	Tomato	Bundaberg	2015	73	5.23	4421	312,487	64.68	this study
BRIP 63666	QEZK01	xp	Tomato	Bundaberg	2015	59	5.28	4473	312,512	64.68	this study
BRIP 62389	QEZR01	xp	Tomato	South Turramurra	2013	70	5.27	4466	225,394	64.68	this study
Xp91–118	CP019725.1	xp	Tomato	USA	2004	1	4.89	4450	4,898,349	65	Potnis et al. 2011
Xp5–6	JZVA01	xp	Tomato	USA	2006	70	5.3	4501	202,262	64.6	Schwartz et al. 2015
Xp7–12	JZVB01	xp	Tomato	USA	2006	65	5.12	4343	207,802	64.9	Schwartz et al. 2015
Xp9–5	JZVD01	xp	Tomato	USA	2006	113	5.38	4624	193,597	64.6	Schwartz et al. 2015
Xp10–13	JZVE01	xp	Tomato	USA	2006	78	5.17	4410	158,830	64.8	Schwartz et al. 2015
Xp11–2	JZVF01	xp	Tomato	USA	2006	50	5.35	4568	302,422	64.6	Schwartz et al. 2015
Xp15–11	JZVG01	xp	Tomato	USA	2006	63	5.31	4520	236,137	64.6	Schwartz et al. 2015
GEV839	JZVK01	xp	Tomato	USA	2012	109	5.44	4669	107,802	64.4	Schwartz et al. 2015
Xp3–15	JZUY01	xp	Tomato	USA	2006	93	5.46	4692	226,886	64.4	Schwartz et al. 2015
Xp8–16	JZVC01	xp	Tomato	USA	2006	62	5.26	4469	241,328	64.6	Schwartz et al. 2015
Xp4–20	JZUZ01	xp	Tomato	USA	2006	71	5.29	4515	250,796	64.7	Schwartz et al. 2015
Xp17–12	JZVH01	xp	Tomato	USA	2006	84	5.2	4387	201,083	64.7	Schwartz et al. 2015
Xp18–15	JZVI01	xp	Tomato	USA	2006	78	5.35	4568	178,519	64.6	Schwartz et al. 2015
GEV909	JZVO01	xp	Tomato	USA	2012	71	5.14	4388	128,792	64.9	Schwartz et al. 2015
GEV940	JZVS01	xp	Tomato	USA	2012	98	5.14	4384	118,066	64.9	Schwartz et al. 2015
GEV968	JZVT01	xp	Tomato	USA	2012	96	5.14	4392	114,913	64.9	Schwartz et al. 2015
GEV1001	JZVV01	xp	Tomato	USA	2012	78	5.18	4402	123,606	64.8	Schwartz et al. 2015
GEV872	JZVL01	xp	Tomato	USA	2012	80	5.14	4401	143,709	64.9	Schwartz et al. 2015
GEV893	JZVM01	xp	Tomato	USA	2012	116	5.13	4387	105,783	64.9	Schwartz et al. 2015
Xp4B	JZUX01	xp	Tomato	USA	1998	214	5.22	4469	73,796	64.6	Schwartz et al. 2015
GEV904	JZVN01	xp	Tomato	USA	2012	134	5.14	4405	77,145	64.9	Schwartz et al. 2015
GEV936	JZVR01	xp	Tomato	USA	2012	104	5.14	4390	111,234	64.9	Schwartz et al. 2015
GEV915	JZVP01	xp	Tomato	USA	2012	70	5.12	4356	180,795	64.9	Schwartz et al. 2015
GEV917	JZVQ01	xp	Tomato	USA	2012	122	5.14	4390	85,814	64.9	Schwartz et al. 2015
GEV1054	JZVY01	xp	Tomato	USA	2012	85	5.23	4450	128,850	64.7	Schwartz et al. 2015
GEV1044	JZVX01	xp	Tomato	USA	2012	82	5.24	4456	128,850	64.7	Schwartz et al. 2015
TB6	JZWA01	xp	Tomato	USA	2013	148	5.27	4473	249,903	64.6	Schwartz et al. 2015
TB9	JZWB01	xp	Tomato	USA	2013	268	5.32	4483	236,007	64.6	Schwartz et al. 2015
GEV993	JZVU01	xp	Tomato	USA	2012	69	5.15	4385	176,938	64.9	Schwartz et al. 2015
GEV1026	JZVW01	xp	Tomato	USA	2012	70	5.15	4373	158,062	64.9	Schwartz et al. 2015
GEV1063	JZVZ01	xp	Tomato	USA	2012	93	5.16	4384	128,850	64.8	Schwartz et al. 2015
TB15	JZWC01	xp	Tomato	USA	2013	261	5.33	4505	233,353	64.6	Schwartz et al. 2015
Xp2010	JZVJ01	xp	Tomato	USA	2010	13	5.25	4575	1,113,511	64.6	Schwartz et al. 2015
4P1S2	JRWW01	xp	Tomato	Sicily	2011	442	5.51	5216	41,480	64.7	Torelli et al. (Unpublished)
DAR 33341	QEYV01	x sp	Capsicum	Peats Ridge	1979	57	5.18	4387	199,700	64.66	this study
BRIP 62409	QEZJ01	x sp	Tomato	Stanthorpe	2015	37	4.92	4033	210,840	65.92	this study
BRIP 62411	QEZI01	x sp	Tomato	Stanthorpe	2015	46	4.81	4002	334,519	66.14	this study
BRIP 62415	QEZH01	x sp	Tomato	Stanthorpe	2015	49	4.85	3983	325,857	66.06	this study
BRIP 62418	QEZG01	x sp	Tomato	Stanthorpe	2015	67	5.09	4274	193,024	65.8	this study
BRIP 38864	QEZA01	xv	Wild tomato	Bowen	1982	69	5.22	4439	377,679	64.23	this study
DAR 26931	QEYU01	xv	Capsicum	Burrundulla	1977	46	5.18	4386	243,089	64.25	this study
DAR 26933	QEYT01	xv	Capsicum	Redland bay	1977	1851	4.93	4899	4596	64.09	this study
BRIP 62388	QEZD01	xv	Tomato	South Turramurra	2012	85	5.31	4488	142,449	64.02	this study
BRIP 62413	QEZF01	xv	Tomato	Stanthorpe	2015	56	5.37	4574	377,588	64.12	this study
BRIP 62423	QEZE01	xv	Tomato	Stanthorpe	2015	69	5.37	4582	467,775	64.12	this study
BRIP 62428	QEZC01	xv	Tomato	Tenterfield	2015	66	5.36	4575	298,833	64.12	this study
BRIP 62429	QEZB01	xv	Tomato	Tenterfield	2015	60	5.36	4567	198,567	64.13	this study
BRIP 38861	QEYZ01	xv	Tomato	Victoria Point	1981	46	5.23	4442	377,697	64.22	this study
ATCC 35937	AEQV01	xv	Tomato	New Zealand	1955	296	5.53	4768	41,380	64.1	Potnis et al. 2011
53 M	JSYJ01	xv	*Capsicum annuum* cv. Kurtovska kapija	Macedonia	2005	344	5.29	4526	29,340	64	Vancheva et al. 2015
15b	JSXZ01	xv	Capsicum annum	Bulgaria	2005	338	5.29	4517	31,449	64	Vancheva et al. 2015
LMG 919	JTEF01	xv	Tomato	Zimbabwe	na	420	5.12	4391	21,731	64.3	Lefeuvre et al. (Unpublished)
LMG 920	JTEG01	xv	Tomato	Italy	1956	326	5.16	4430	28,850	64.2	Lefeuvre et al. (Unpublished)

^a^Brisbane pathology herbarium ID

^b^WGS project number or Genbank ID where complete genome is available

^c^*xa X. arboricola*, *xe X. euvesicatoria*, *xp X. perforans*, *xv X. vesicatoria*, *x sp Xanthomonas* sp.

^d^xa pv ce: *X. arboricola* pv. *celebensis*, xa pv j: *X. arboricola* pv. *juglandis*, xa pv co: *X. arboricola* pv. *corylina*, xa pv p: *X. arboricola* pv. *pruni*

^e^Data not available

^f^CDS as determined by the annotation of this study for consistency

Included statistics are contig number, length in Mbp, CDS, N50 and GC content. All reported CDS numbers were generated in this study. The metadata for Australian strains was sourced from Roach et al. 2017

**Fig. 1:**
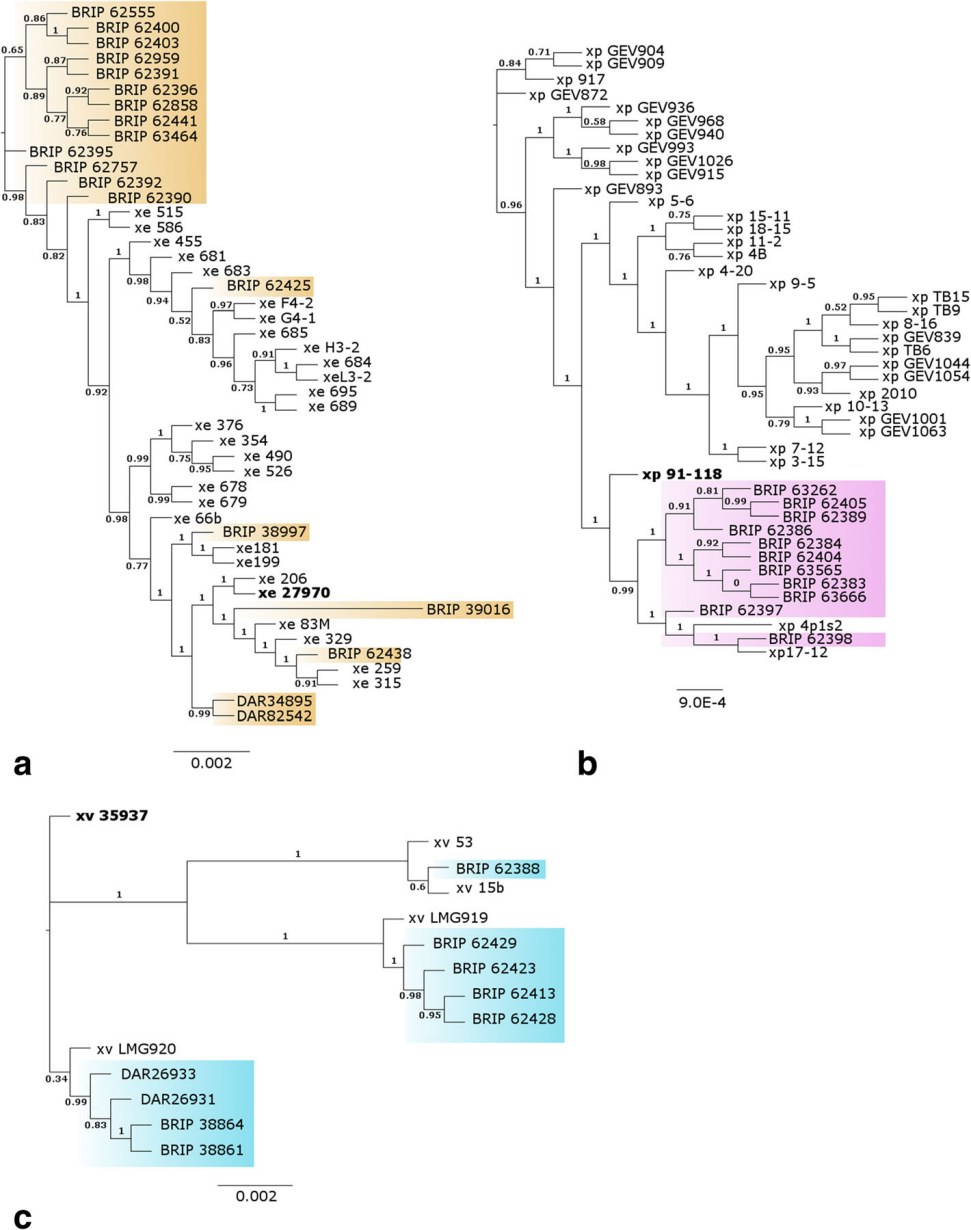
Phylogeny of Australian and Genbank genomes of **a**) *X. euvesicatoria*, **b**) *X. perforans* and **c**) *X. vesicatoria* based on core genome alignments generated by the Roary program. Australian strains are indicated by BRIP and DAR collection prefixes and highlighted; all others are public genomes of related species. Type strains are indicated in bold and branch support values are displayed to clade level (measured with the Shimodaira-Hasegawa test within FastTree). Branch length is indicated by the scale bar

The SNP-based phylogenetic tree arranges most strains in this study into distinct clades (Additional file 1: Figure S1). The *X. euvesicatoria* and *X. perforans* clades grouped distinctly from the *X. vesicatoria*, *X. gardneri* and *X. arboricola* clades. The *X. vesicatoria* and *X. arboricola* clades contain three and four distinct subclades respectively (branch support values of 1). Four strains from tomato in Stanthorpe (BRIP 62409, 62411, 62415, and 62418, designated the uncharacterised *Xanthomonas* sp. clade) resolved in a clade distinct from its closest relative *X. arboricola*. The core genome phylogenies of individual BLS-causing species (excluding *X. gardneri*, *X. arboricola* and the uncharacterised *Xanthomonas* clade) clustered Australian strains in clades with multiple strains from other countries (Fig. 1). Australian strains of *X. perforans* cluster with overseas isolates xp 91–118, xp 4p1s2 and xp 17–12. Nine Australian *X. euvesicatoria* strains cluster in a clade by themselves, with the other ten distributed across clades with overseas strains. Australian *X. vesicatoria* strains cluster in three clades with overseas strains, distant from the type strain ATCC 35937.

All *X. euvesicatoria* strains apart from BRIP 39016 tested were pathogenic on both capsicum [4] and tomato, where BRIP 39016 was determined to be non-pathogenic on both hosts. Strains of *X. vesicatoria* and *X. perforans* were pathogenic on tomato as determined previously [4], and non-pathogenic on capsicum. Strains of the uncharacterised *Xanthomonas* sp. were non-pathogenic on both hosts, and strains of *X. arboricola* were designated non-pathogenic on tomato [4] and capsicum. Pathogenicity of *X. euvesicatoria* on tomato was observed as small, dark lesions with yellow halo that displayed bacterial streaming. Isolations resulted in yellow, gram negative colonies.

**Fig. 2:**
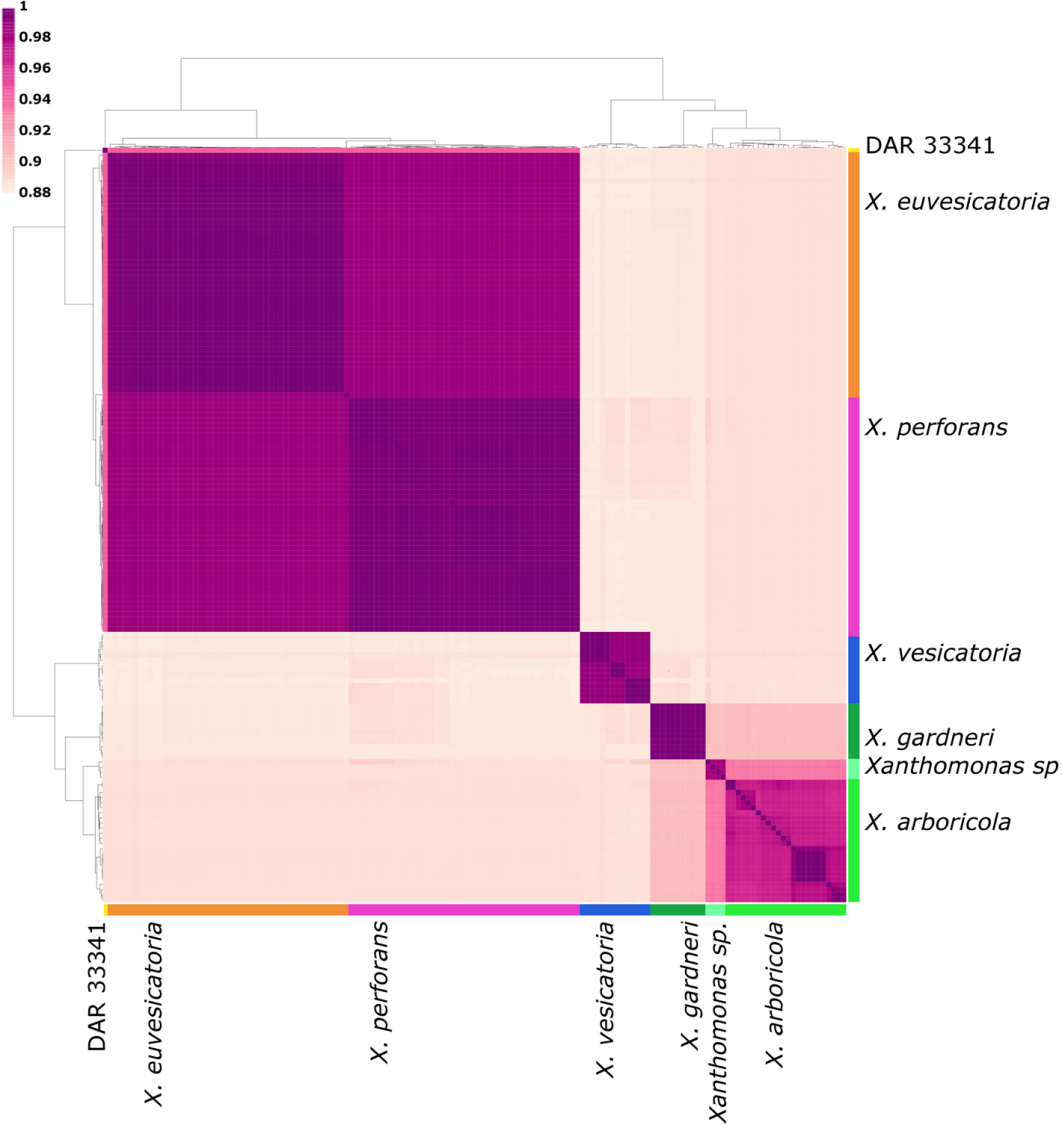
A heat map and dendrogram of average nucleotide identity (ANI) between 147 *Xanthomonas* genomes. The coloured bars represent the species as indicated in the SNP phylogeny and supported ANI values shown here. *Xanthomonas perforans* strains of *X. euvesicatoria* are indicated separately to highlight ANI differences. ANI is depicted as the colour gradient indicated by the legend: darker = 1 (100% ANI), lighter = 0.88 (88% ANI)

Average nucleotide identity (ANI) of core genome sequence analysed in this study are presented in Fig. 2. An ANI of 93% supports the separation of *X. arboricola* and the four uncharacterised *Xanthomonas* sp. strains into two separate species. ANI values of > 98% showed the genetic similarity of *X. perforans* and *X. euvesicatoria* while also displaying conserved separation. ANI analysis indicates that BRIP 39016 and DAR 26930 are also strains of *X. euvesicatoria* (ANI > 98%). Strain DAR 33341 has an ANI < 95% to all other strains in the analysis and 94% to *X. euvesicatoria* and was therefore excluded from *X. euvesicatoria*.

### Pan genome composition

**Fig. 3:**
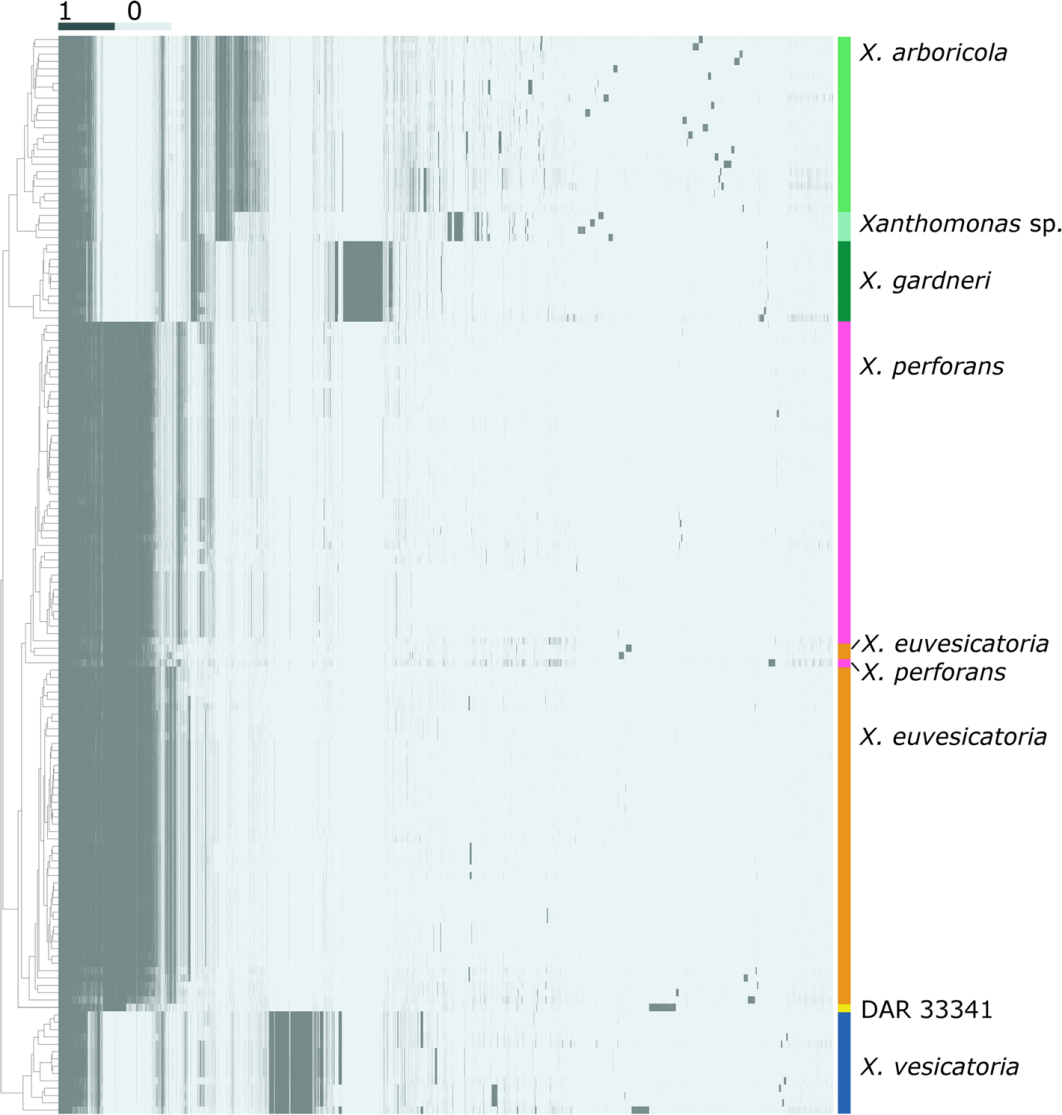
Cluster matrix of 147 *Xanthomonas* genomes with dendrogram based on homologue presence (dark) and absence (light). Species groupings are indicated with coloured bars as determined by phylogeny and ANI. *Xanthomonas perforans* strains of *X. euvesicatoria* are indicated separately to highlight homologue differences. The four Australian strains most closely related to *X. arboricola* are designated in the text as an uncharacterised *Xanthomonas* species

**Fig. 4:**
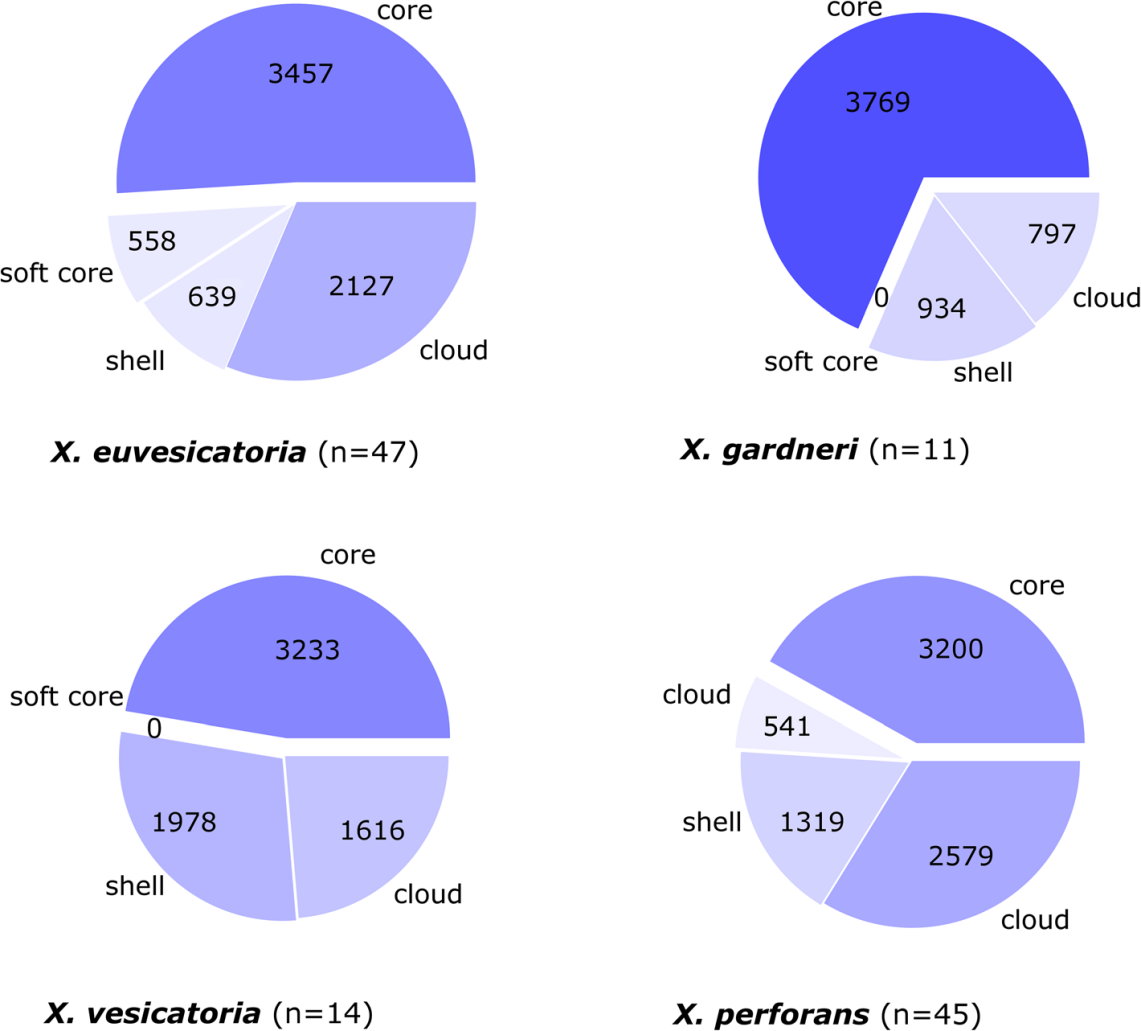
Pie plots of gene content in core, soft core, shell and cloud genomes describing the pan genome for *X. euvesicatoria, X. perforans, X. vesicatoria* and *X. gardneri*. The core genome is defined as genes present in 99–100% of strains; soft core, shell and cloud genomes are defined as 95–99%, 15–95% and 0–15% respectively. Number of genomes in each pan genome is indicated as ‘n’

The nucleotide homologue cluster matrix grouped all strains (Fig. 3) in a generally similar topology to the phylogeny while also highlighting distinct differences between species. All species contained unique homologues (280 in *X. arboricola*, 70 in *X. euvesicatoria*, 69 in *X. perforans*, 416 in the combined *X. euvesicatoria* and *X. perforans* clades, 1639 in *X. gardneri*, 1646 in *X. vesicatoria*, and 609 in the uncharacterised *Xanthomonas* sp. clade). Pan genome pie charts (Fig. 4) based on the homologue matrix (Fig. 3) describe the core, soft core, shell and cloud genome content of each species. Gene discovery plots for each species (Additional file 2: Figure S2) showed that the number of new genes approached zero as genome number increased.

### Predicted and isolated plasmids and predicted effector content

Contigs originating from plasmids were assembled for 48 of 50 sequenced Australian strains, resulting in a total of 61 plasmids reconstructed for 41 strains (Table 2). A 31 kb plasmid (13 plasmids of 31,328 bp and two of 31,318 bp) was present in 15 of the *X. euvesicatoria* strains studied, although it was not found in any *X. perforans* strains. A 150 kb plasmid (six plasmids of 159,114 bp and one of 159,115 bp) was present in seven *X. euvesicatoria* strains and none of the *X. perforans* strains. An 88 kb plasmid (3 plasmids of 88,047 bp, 88,057 bp, 88,063 bp) was present in three of the *X. vesicatoria* strains. A 47 kb plasmid (three plasmids of 47,218 bp, one of 47,214 bp) was present in four strains of *X. vesicatoria*. The remaining plasmids were not unique to individual species. A 17 kb plasmid (two of 17,360 kb, one of 17,359 kb, 17,382 kb, and 17,377 kb) was present in four *X. euvesicatoria* strains and one *X. arboricola* strain. Plasmids of 40–41 kb were present in two *X. euvesicatoria* strains, one *X. perforans* strain and two *X. vesicatoria* strains. A single *X. euvesicatoria* strain contained two plasmids of 28,462 kb and 32,900 kb, while a strain of the uncharacterised *Xanthomonas* sp. contained a plasmid of 28,836 kb. The 19 plasmids less than 10 kb in size were found in five *X. euvesicatoria* strains, nine strains of *X. perforans*, one strain of the uncharacterised *Xanthomonas* clade, and DAR 33341. Protein sequence with homology to established effectors were detected in reconstructed plasmid sequence. Homologues of AvrBsT were detected in each of seven strains of *X. vesicatoria* (four 47 kb plasmids and three 88 kb plasmids); AvrBs3 in two strains of *X. euvesicatoria* on 40 kb plasmids and XopH in an *X. perforans* strain on a 41 kb plasmid.

**Table 2:** Reconstructed circular sequence assembled for Australian *Xanthomonas* draft genomes from whole genome sequence data

Strain	Species ^a^	Plasmid number ^b^	Plasmid .0 ^c^	Plasmid .1	Plasmid .2	Plasmid .3
BRIP 62410	xa	0				
BRIP 62412	xa	0				
BRIP 62414	xa	0				
BRIP 62416	xa	1	17,377			
BRIP 62432	xe	1	17,382			
BRIP 62441	xe	1	31,328			
BRIP 62555	xe	1	31,328			
BRIP 62757	xe	2	159,114	31,328		
BRIP 62858	xe	3	8247	159,114	31,328	
BRIP 62959	xe	1	159,115			
BRIP 38997	xe	2	17,360	***40,439		
BRIP 63464	xe	2	159,114	31,328		
BRIP 39016	xe	2	32,900	28,462		
BRIP 62396	xe	2	31,328	4316		
BRIP 62400	xe	1	31,328			
BRIP 62403	xe	2	31,328	159,114		
BRIP 62390	xe	2	159,114	31,328		
BRIP 62391	xe	2	8247	31,328		
BRIP 62392	xe	1	31,328			
BRIP 62395	xe	1	31,328			
BRIP 62438	xe	0				
BRIP 62425	xe	3	31,328	159,114	17,360	
DAR 34895	xe	1	31,318			
DAR 82542	xe	4	2587	17,350	***40,429	31,318
DAR 26930	xe	1	8201			
BRIP 62383	xp	1	1418			
BRIP 62384	xp	1	2808			
BRIP 62398	xp	1	7812			
BRIP 62404	xp	1	2808			
BRIP 62397	xp	1	**41,842			
BRIP 62386	xp	1	2808			
BRIP 62405	xp	2	2589	5031		
BRIP 63262	xp	2	2589	5031		
BRIP 63565	xp	1	2808			
BRIP 63666	xp	1	1418			
BRIP 62389	xp	0				
BRIP 62409	x sp	0				
BRIP 62411	x sp	1	28,836			
BRIP 62415	x sp	0				
BRIP 62418	x sp	1	1418			
DAR 33341	x sp	2	7154	4921		
BRIP 38864	xv	2	*88057	41,641		
BRIP 62388	xv	0				
BRIP 62413	xv	1	*47218			
BRIP 62423	xv	1	*47218			
BRIP 62428	xv	1	*47218			
BRIP 62429	xv	1	*47214			
BRIP 38861	xv	2	*88063	41,641		
DAR 26931	xv	1	*88047			
DAR 26933	xv	0				

^a^species as determined by whole genome SNP phylogeny and ANI

^b^circular sequences assembled by recycler for each strain

^c^circular sequence ID is indicated by decimal value after strain ID

Length is described in base pairs. * indicates the predicted plasmid contains AvrBsT, ** XopH, ***AvrBs3

Predicted plasmids were investigated by isolating and visualising plasmid DNA of strains BRIP 38864, BRIP 62858, BRIP 62416, BRIP 62423, BRIP 62388, BRIP 62397, BRIP 63464, BRIP 38997 and the extraction control DC 3000. (Additional file 3: Figure S3). Bands approximately of the size predicted were observed in strains BRIP 38864, BRIP 62858, BRIP 62423, BRIP 62397, BRIP 63464 and DC 3000. Where no plasmids were predicted for strain BRIP 62388, bands similar to those of other extractions were observed. For strains BRIP 62416, no plasmids were recovered despite the prediction of 17 kbp circular sequence. BRIP 62423, BRIP 62397, BRIP 38997, and BRIP 62858 may have additional large bands that could not be separated effectively. Sizing is only approximate due to the possibility of multiple plasmid structures (nicked circular sequence, linear sequence, supercoiled plasmids) migrating through the gel at different rates.

### Effector and CAZyme content

**Fig. 5:**
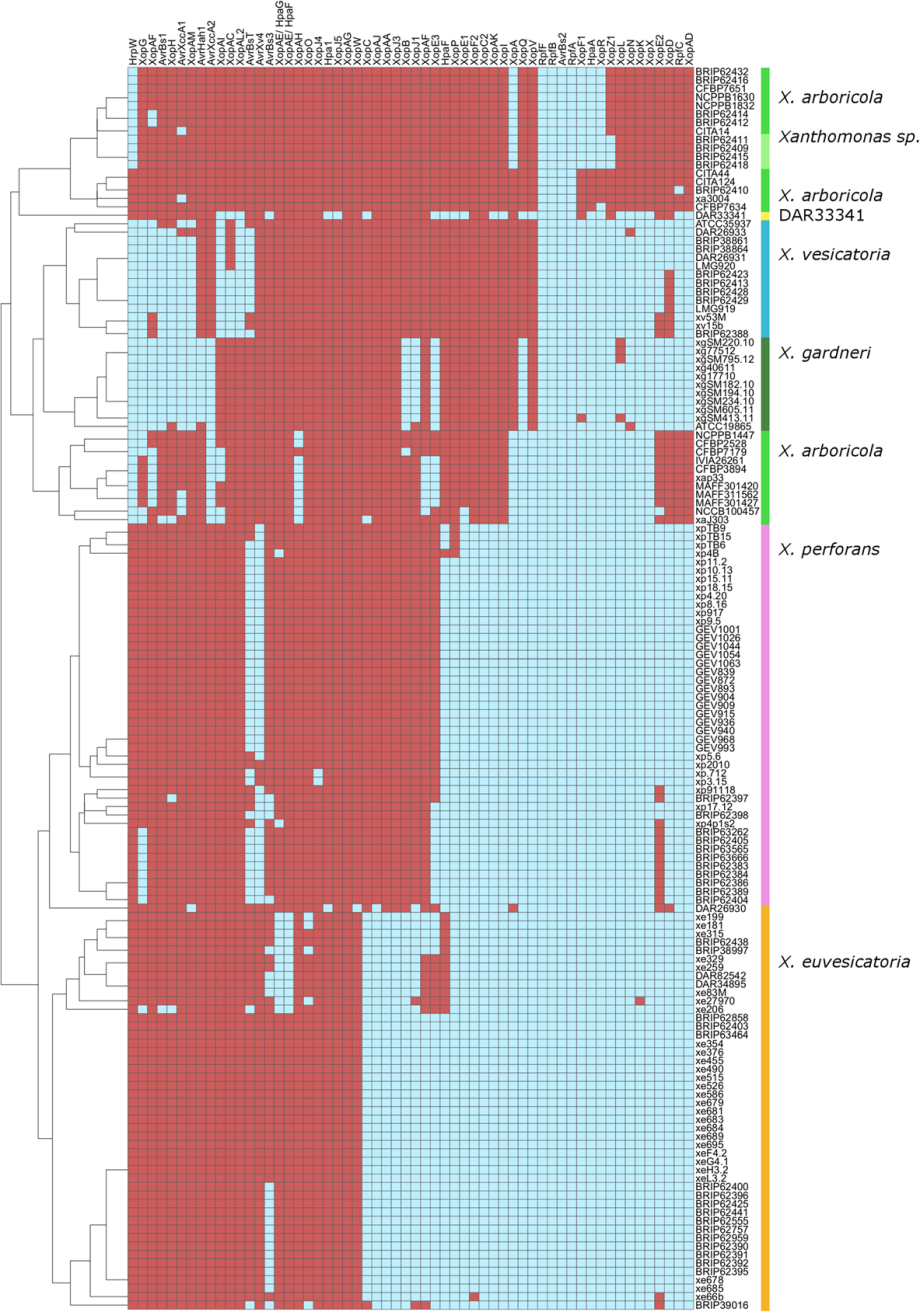
Presence/ absence matrix with dendrogram of effectors identified in 147 *Xanthomonas* genomes. Effector presence is indicated by colour as described in the legend (presence = blue, absence = red). Names and Genbank numbers of identified effectors are listed vertically. Species groupings as determined by phylogeny and ANI are indicated by the horizontal coloured bar as follows: *X. euvesicatoria*; orange, *X. perforans*; pink, *X. vesicatoria*; blue, *X. gardneri*; dark green, *X. arboricola*; green, *Xanthomonas* sp.; light green

The effector profiles of the dataset (Fig. 5) grouped species in the same general topology as the whole genome SNP phylogeny (Additional file 1: Figure S1). Effectors that were core to each species and the entire dataset, as well as effectors discussed in other studies, are listed in supplementary material (Additional file 4: Table S1). The occurrence of important effectors identified in previous studies [1, 17] are also listed here. Retained as core to BLS-causing species (*X. euvesicatoria*, *X. perforans*, *X. vesicatoria* and *X. gardneri*) as listed previously (AvrBs2, XopR, XopX, XopZ1, XopAD, XopN, XopF1, XopK, XopL, XopQ, XopD) [1] are AvrBs2, XopR, XopX, XopZ1 and XopAD. Several of these effectors previously considered core were detected in all but a few strains of certain species; XopN was absent in one *X. vesicatoria* and one *X. gardneri* strain, XopF1 was absent in one *X. gardneri* strain, XopK was absent in one *X. euvesicatoria* strain, and XopL was absent in four *X. gardneri* strains. XopQ was absent in all *X. vesicatoria* strains and XopD was absent in eight *X. vesicatoria* strains.

**Fig. 6:**
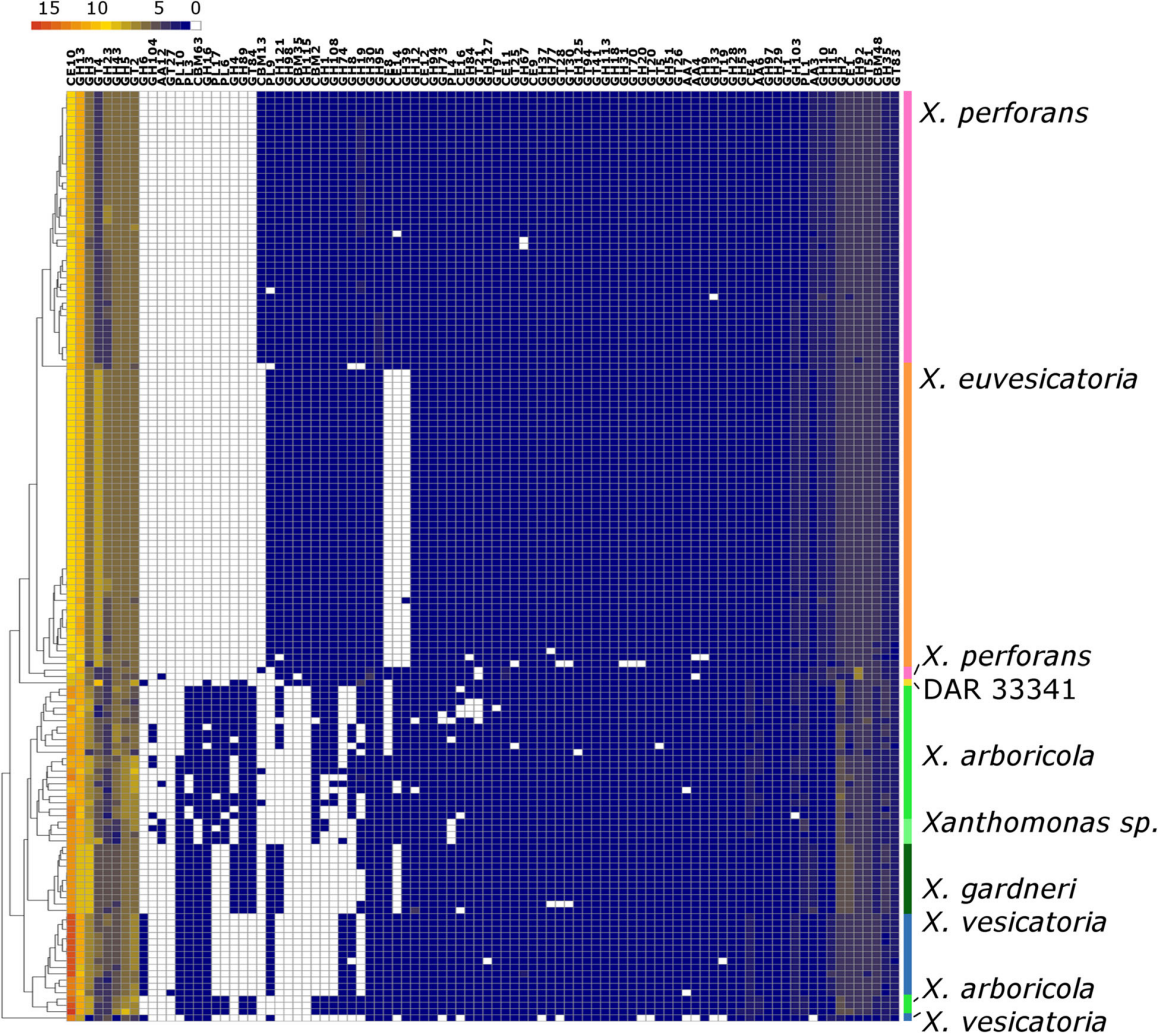
Cluster matrix and dendrogram based on number of CAZyme families identified in 147 *Xanthomonas* genomes. Number of CAZyme families present in each strain is indicated by the red-blue scale of the figure legend (17 families = red, 1 = blue, 0 = white). CAZyme families are listed vertically. Horizontal coloured bars represent species as indicated by SNP phylogeny and ANI. *Xanthomonas perforans* strains of *X. euvesicatoria* are indicated separately to highlight differences in cazyme profile

The dendrogram based on CAZyme family data (Fig. 6) grouped species distinctly in the same general topology as seen in the genome SNP phylogeny (Additional file 1: Figure S1). In contrast to the SNP phylogeny, the CAZyme dendrogram clusters some *X. arboricola* and *X. perforans* strains outside of their group. A total of 92 carbohydrate active and facilitator enzyme families were identified, revealing groups present or absent in certain species and clades (Fig. 6, Additional file 5: Table S2). CAZyme families of cell wall degrading enzyme genes identified in BLS-causing *Xanthomonas* by Potnis et al. [27] were also identified in this dataset (Additional file 5: Table S2).

## Discussion

Genomic analysis of BLS-associated *Xanthomonas* strains revealed diverse groups with distinguishing features that will have implications for future pathogenicity and taxonomic studies. Phylogenetic analysis (SNP, core genome, ANI) supports the close relationship between *X. euvesicatoria* and *X. perforans*. An uncharacterised *Xanthomonas* species (BRIP 62409, BRIP 62411, BRIP 62415 and BRIP 62418) was demonstrated to be distinct from closely related strains of *X. arboricola* according to the SNP phylogeny and ANI. The effector and CAZyme profiles of species that differ in pathogenicity displayed clear differences that may reflect differences in epidemiology and niche survival.

### Taxonomic status of BLS-causing *Xanthomonas* species

The phylogenies and homologue matrix generated in this study support the current taxonomic status of *X. euvesicatoria*, *X. perforans* and *X. vesicatoria* [28], also confirming previous findings [4] that *X. gardneri* was not detected in Australian strains of this study. A recent study based on whole genome ANI determined that *X. euvesicatoria* and *X. perforans* were not genetically distinct enough to be considered separate species [29]. Our study found an ANI of 98.6% between strains of *X. euvesicatoria* and *X. perforans*, supporting these findings. While genetically very similar, strains of *X. perforans* were still clearly distinguished from *X. euvesicatoria* and other species in the phylogenetic analyses and the analyses of secretion systems. This may reflect differences in phenotype and pathogenicity, as Australian strains of *X. euvesicatoria* and *X. perforans* are generally isolated from capsicum and tomato respectively [4]. This example of genetically similar species being reliably differentiated by other measures is important to consider in the ongoing debate of how to classify bacteria, as a name could reflect phylogenetic groupings or phenotypic (and pathogenic) differences.

The core genome phylogenies of the individual BLS-causing species *X. euvesicatoria*, *X. perforans* and *X. vesicatoria* cluster Australian strains in multiple clades with strains from overseas (Fig. 1). Australian *X. vesicatoria* strains are similar to strains from Italy (LMG 920), Zimbabwe (LMG 919), Macedonia (53 M) and Bulgaria (15b). Australian strains of *X. perforans* generally resolved in a clade of their own, closely related to the type strain. A subclade clustered BRIP 62398 and BRIP 62397 with xp 4p1s2 and xp 17–12, two strains from Sicily and the USA respectively (Table 1). Australian strains of *X. euvesicatoria* generally clustered in their own clade, with strains BRIP 62425, BRIP 38997, BRIP 39016, BRIP 62438, DAR34895 and DAR82542 dispersed throughout the phylogeny with overseas strains, generally from the USA. The presence of Australian and overseas strains together in different clades may represent direct introductions of pathogens or the general distribution of the species across the world over time.

### Pan genome of collected *Xanthomonas* species

The homologue matrix of strains in this study reflected the whole genome SNP phylogeny, while also highlighting blocks of unique and shared regions containing hundreds of genes that may be relevant to host specificity, virulence, other phenotypic traits and niche adaptations. This matrix shows that the genomes of *X. euvesicatoria* strains BRIP 39016 and DAR 26930 were included in the *X. perforans* group, indicating there may be some recombination events present in these strains/ species. The Australian strains were not significantly differentiated from overseas strains by this matrix, indicating a certain level of species homogeneity distributed across the world.

The core genomes of species in this dataset represent conserved functionally essential genes, while the larger accessory and cloud genomes contained genes that may be specific to growth or pathogenicity, particularly as species with different pathogenic capabilities are present in the homologue matrix. Most species analysed individually reflected this trend of large accessory genomes, with the exception of *X. gardneri*, which was influenced by the small sample size of highly similar strains. The gene discovery plots for the pan genomes of these species showed that the genomes of *X. euvesicatoria* and *X. perforans* can be considered closed. Plotting gene discovery indicates there is some potential that additional genomes would result in detection of new genes for *X. vesicatoria* and *X. gardneri*. The large accessory genomes observed in many of these species reflects the genetic diversity seen in other studies of *Xanthomonas* species [3], and also suggests that genome plasticity could result in new genes being added to the population.

### Predicted plasmids reconstructed from collected *Xanthomonas* strains

The seven 150 kb plasmids from *X. euvesicatoria* had high homology to the *X. euvesicatoria* plasmid pLMG930.2 of similar size (167,496 bp). Similarly, the 31 kb group of Australian plasmids found in *X. euvesicatoria* shared homology with *X. euvesicatoria* and X. citri plasmids pLMG930.4 (GenBank, unpublished) and CFBP6167 plasmid pG [7]. This homology, and their presence in *X. euvesicatoria* strains (notably absent in *X. perforans* strains) indicates these 150 kb and 31 kb sequences are previously characterised plasmids. This is likely also the case for plasmids of 47 kb found in *X. vesicatoria*, as they are similar in size and homology to the *X. vesicatoria* plasmid pLMG911.2 (CP018727.1, 47 kb). By contrast, three 88 kb plasmids in *X. vesicatoria*, although similar by sequence homology, are much smaller than the *X. vesicatoria* plasmid pLMG911.1 (192,558 bp).

Plasmids of 40 kb found in *X. vesicatoria*, *X. euvesicatoria* and *X. perforans* were most similar to previously reported plasmids of *X. campestris* pv. *campestris* strains CN14 (GenBank: CP017318.1) and CN15 (CP017325.1), and *X. perforans* pLH3.3 (NZCP018474.1) and pLH3.2 (NZCP018476.1), all of varying sizes. Three plasmids of approximately 28–32 kb were slightly different in size and homology to the 31 kb plasmids, their presence in older and uncharacterised *Xanthomonas* strains indicating they may be more distantly related. Interestingly, five 17 kb plasmids of *X. euvesicatoria* did not significantly match any plasmid sequence and were not recovered in the plasmid isolations of BRIP 62416 and BRIP 38997. Evidence for large (80–150 kbp) plasmids was observed in the plasmid extractions, as well as bands that are likely 30–40 kbp in size (Additional file 3: Figure S3). No definitive bands at 8 kbp or 17 kbp were observed in strains BRIP 62858, BRIP 62416 and BRIP 38997, indicating some smaller plasmids may be a result of computational reconstruction. There also appeared to be some plasmids present that were not detected by the selected programs, as in BRIP 62388. False positives and negatives may be a result of integrative conjugative elements or repeat regions that may require further sequencing to fully resolve.

Avirulence genes have been found in many described plasmids of most genera, including *Xanthomonas* pathogens [8]. Sequence with homology to three effectors (AvrBs3, AvrBsT and XopH) were detected in assembled plasmids of ca. 40–47 kb. AvrBsT was detected in most *X. vesicatoria* plasmids (seven of nine), and has been known to exist on plasmids since its characterisation [9, 30]. As in other studies of plasmid-borne effectors [31], the presence of effectors here demonstrates these circular elements have the potential to influence pathogenicity. XopH, detected in one plasmid of *X. perforans*, has been suggested as a potential determinant of pathogenicity in *X. arboricola* pv. *corylina* [32]. It has also been found in *X. campestris* pv. *campestris* [33], and here was found in the majority of *X. gardneri* and *X. vesicatoria* chromosomes. Other genes, for example copper tolerance genes, have been found on *Xanthomonas* plasmids [8], suggesting other significant adaptive genes in addition to effectors may be investigated in future studies.

### Effector profiles of *Xanthomonas*

Many studies have presented core effector lists for *Xanthomonas* pathogens and found that these effectors are integral to certain strains/species and play key roles in pathogenicity [1, 13, 17, 29, 34]. The effector profiles determined by this study show distinct patterns specific to species and clades. We have revised the core and specific effector list for *Xanthomonas* species causing BLS and contrast them with species displaying different pathogenic abilities.

### Core and specific effectors

Few effectors were found to be shared between closely related phylogenetic groups, a finding consistent with a previous study on the type strains of four BLS-causing *Xanthomonas* species [27]. Subsequent studies have noted that strains may display different effector profiles to that of the type strain of their species [17], a pattern also observed in this study. The core effectors previously identified in the type strains of the BLS pathogens *X. gardneri*, *X. perforans*, *X. vesicatoria* and *X. campestris* pv. *vesicatoria* (*X. euvesicatoria*) [27] were expanded [17] with the addition of XopE2 and a member of the YopJ family (AvrBsT and XopJ1). Barak et al. [29] further refined the list of core effectors, finding all effectors previously identified [27], with the exception of XopAD that displayed internal stop codons in some *X. euvesicatoria* strains. The analysis of effectors in species that do not cause BLS provides an opportunity to compare and contrast effector profiles with *X. vesicatoria*, *X. euvesicatoria* and *X. perforans*. The *Xanthomonas* sp. clade has few effectors, most of which are shared with some *X. arboricola* and *X. vesicatoria* strains. Reduced T3SS effector repertoires do not necessarily indicate a lack of pathogenic capability [35], however it is likely these effectors (Additional file 4: Table S1) are not directly involved in pathogenicity on tomato or pepper due to their presence in non-pathogenic strains. The Australian *X. arboricola* strains in this study have varied effector profiles with few common effectors. As there are relatively few sequenced strains of *X. arboricola* from different hosts, it is difficult to draw meaningful conclusions about effector profiles in relation to their pathogenicity. The variation observed in these profiles is likely a result of wider host range, presenting a point of contrast to the other groups.

In this study, the core effectors AvrBs2, XopAD, XopR, XopX and XopZ1 were found in the majority of strains of *X. euvesicatoria*, *X. gardneri*, *X. perforans* and *X. vesicatoria*. All of these strains together with those of *X. arboricola* and the uncharacterised *Xanthomonas* sp. clade contained rpfA, rpfB and rpfF, members of the rpf gene family that regulate pathogenicity factors and biofilm production [36]. Homologues of AvrBs2, involved in the modulation of effector delivery [37] were also found in all strains of this study. Other effectors previously listed as core to BLS-causing *Xanthomonas* species were detected in most strains of these species with some exceptions as listed above. Interestingly, many of these effectors are also present in *X. arboricola* strains and the uncharacterised *Xanthomonas* clade. For example, XopF1 was only absent in one strain of *X. gardneri*, but was detected in BLS-causing species as well as most *X. arboricola* and all strains of the uncharacterised clade. The core effectors XopK, XopL and XopN, were also found in strains not isolated from tomato or pepper, which indicates these proteins may not be associated with specificity to these hosts. No single effector in this study appeared to be consistently associated with pathogenicity on tomato based on comparison with the *X. arboricola* and *Xanthomonas* spp. clades. This was also the case for pepper pathogenic strains, though XopAA and XopJ3 were present only in the pepper pathogenic *X. euvesicatoria* and the non- pepper pathogenic BRIP 39016. The profiles presented here represent homologues in predicted protein sequence, so it is possible inactivation in effector gene sequences play a role in pathogenicity as well.

As demonstrated by Barak et al. [29], the core effectors listed above do not determine pathogenicity on tomato due to their presence in an *X. euvesicatoria* strain isolated from rose. One particular clade of *X. arboricola* (containing the MAFF strains) shared many effectors with BLS-causing species, further emphasising the need for comprehensive pathogenicity studies to tie effector profile to functional traits.

### Effectors and host range of the *X. euvesicatoria* and *X. perforans* clades

While *X. euvesicatoria* is commonly reported as a pathogen of tomato and pepper, all but two *X. euvesicatoria* strains (BRIP 39016 and DAR 26930) from Australian crops were found in capsicum and chilli [4]. Recent reports indicate it is more common to observe *X. perforans* (and *X. gardneri*) in tomato and *X. euvesicatoria* in peppers [17, 38, 39]. Prior to 1991, *X. euvesicatoria* was the main BLS pathogen on tomato in Florida [1]. This indicates it was once more common to find *X. euvesicatoria* in tomato than it is today. As the only Australian *X. euvesicatoria* strains isolated from tomato were from 1973 and 1976, Australian *X. euvesicatoria* populations reflect this host shift observed overseas. One *X. perforans* strain (Xp2010) from Florida displayed dual infecting ability in pepper and tomato [27]. An Australian strain, BRIP 62398, phylogenetically related to Xp2010 did not share this trait, as all tested Australian *X. perforans* strains were pathogenic only on tomato. While this indicates pathogenicity traits are not necessarily reflected in phylogenies, variation in virulence on pepper of certain phylogenetic groups has been noted [17].

Strains of *X. euvesicatoria* and *X. perforans* are genetically similar and share a similar effector profile, while still displaying notable differences. The core effectors XopF1, XopL, XopN, XopQ, XopR, XopX, XopAK, were conserved in the *X. euvesicatoria* and *X. perforans* strains in this study as well as in a previous study (that did not include *X. perforans* strains) [29]. While core effectors may indicate evolutionary history, several studies note that functionality of effector genes must be investigated in addition to presence or absence [17, 29]. Australian strains of *X. euvesicatoria* displayed almost identical profiles to those of overseas strains, apart from a group of 11 that contain an AvrBs3 homologue along with xe678 and xe685 that likely reflect pathogenicity differences. Australian *X. perforans* effector profiles were also similar to other *X. perforans* strains, though they (as well as xp91–118 and xp4p1s2) lack XopE2. Australian *X. perforans* (excluding BRIP 62397) appear to have XopE3 where all other *X. perforans* strains do not. These presences and absences may have pathogenicity implications according to the description of the XopE family [40], though this pattern does also reflect their clade groupings in the core genome phylogeny. XopE3 has also been implicated in citrus pathogenicity [41]. Further investigation into the function of these effectors may reveal the significance of these patterns.

An effector that has been used to track population changes is the 600 amino acid protein XopAE, which is a fusion of the HpaF and hpaG effectors [27]. The majority of Australian *X. euvesicatoria* and *X. perforans* strains contained a XopAE homologue, while four *X. euvesicatoria* strains (BRIP 62438, BRIP 38997, DAR 34895 and DAR 82542) had hpaG and hpaF as separate effectors. These strains were collected from locations and/ or time points different from the rest of the collection, possibly reflecting different introductions or outbreaks. Barak et al. [29] suggested the presence of the translational mutation and the single alleles represented separate introductions, as they observed in strain LMG918. The difference in effector profiles between strains separated by time has also been noted previously [17] and is reflected in historical strains of *X. euvesicatoria* in this study, in particular BRIP 39016 and DAR29630 that are also separated geographically.

### Effectors of *X. vesicatoria* and *X. gardneri*

Strains of *X. vesicatoria* have a distinct effector profile similar to *X. gardneri*, which reflects their position in the whole genome SNP phylogeny. The variation of effector profile within *X. vesicatoria* reflects the phylogenetic clades identified, rather than specific differences in Australian and overseas strains. Homologues of XopAG and XopAI have previously been identified as specific to *X. vesicatoria* [27]. However, we have shown that homologues of XopAG exist in DAR 33341 (*Xanthomonas* sp.) and an *X. arboricola* strain (NCPPB 100457). XopAI was also detected in these and an additional five strains of *X. arboricola*. Previous studies of the *X. gardneri* effector profile found differences between the type and other strains, which is also evident in this study [17].

### Key CAZymes

Similar to the effector profiles, the cazyme profiles grouped strains mostly into species, highlighting regions of difference. No differences between Australian and overseas strains within species were detected. CAZyme genes and families have been identified previously in the type strains of BLS-causing *Xanthomonas* species [27] and reflect the profiles seen in this study. Cellulases are known to be common to the gammaproteobacteria [42], and the abundance of GH families was expected. The xylanase families GH10 and GH30 [25, 43] were present in all strains, indicating these groups share similar strategies for degrading plant cells. Identifying core carbohydrate active enzymes needs further investigation into the proteins and genes of these enzyme families [27]. Carbohydrate utilisation reactions have been used as a diagnostic tool for many years to differentiate bacterial species based on substrate usage [23]. It has also been suggested that secreted cell wall degrading enzymes play a role in host adaptation, with several studies linking these enzymes and pathogenicity [35].

### CAZyme differences between species

The CAZyme family profiles of the *X. euvesicatoria* and *X. perforans* strains were nearly identical, apart from the absence of three families (CE8, CE14, and GH39) in the *X. euvesicatoria* strains. The absence of these families of plant polysaccharides, acetylases and pectinases highlights a distinct difference between these two groups. The absence of CE8 (a pectin methylesterase family) in *X. euvesicatoria* strains correlates with their lack of ability to degrade pectin [25, 43].

The *X. euvesicatoria* strains had mostly glycosyl hydrolase (GH) and polysaccharide lyase (PL) families (PL10, PL3, CBM63, GH16, PL17, PL6, GH4, GH89, GT84) and lacked many CAZyme families present in other species. The CAZyme families PL3, CBM63 and GH16 were found in all *X. vesicatoria* and *X. gardneri* strains and many *X. arboricola* strains. The conserved presence of some CAZyme families that are present or absent in some species may indicate different substrate utilisation capabilities. Groups of cellulose degrading enzymes also display different profiles, which indicates that species have different modes of action on this substrate. The families GH5, GH9 and GH12 were found in most or all of the strains in this study, GH8 and GH6 had a more restricted distribution. In particular, GH6 was identified only in strains of *X. vesicatoria* and DAR 33341, which may reflect different strategies or evolutionary pathways for degrading cellulose.

## Conclusions

This study has provided an overview of the genome structure and content of several *Xanthomonas* species and expanded the original identification of Australian species associated with BLS. We support the taxonomic status of *X. euvesicatoria* and *X. perforans* as one species, though it is clear these strains also have conserved differences that complicate taxonomy. Our analysis of effector proteins and carbohydrate active enzymes links pathogenic data with proteins detected in the genomic analysis, demonstrating that while these profiles differ between species no single pathogenicity factor was identified. It is clear that some differences may also exist in Australian populations regarding effector content. The limitations of bioinformatic reconstruction of plasmids was also highlighted. This study has furthered the understanding of species that cause BLS and provided several points of future study to improve the understanding of Australian bacterial populations.

## Methods

### Isolate collection, pathogenicity testing and sequencing

Strains of *Xanthomonas* spp. associated with BLS in Australia were collected as described in Roach et al. [4]. To determine pathogenicity on tomato and capsicum, all isolates (excluding DAR strains as only genomic data was available) were inoculated on susceptible *Capsicum annuum* var. Yolo Wonder and susceptible *Solanum lycopersicum* var. Grosse Lisse. Overnight cultures of bacteria were diluted in distilled H20 to concentrations of 1 × 10^8^ cfu/ml and sprayed onto plants until run-off. Pathogenicity was recorded after approximately 7 days. Pathogenicity on host of isolation has been reported [4]. Pathogenicity on the alternative host was observed as small, dark lesions with yellow halo that displayed bacterial streaming.

The dataset of 50 Australian strains was comprised of 44 strains held in the Queensland Plant Pathology Herbarium (BRIP) culture collection and six sequenced Australian strains provided by the NSW Plant Pathology Herbarium (DAR) (Table 1). Selected strains of each identified species represented a range of taxa, host and geographical distribution. Strains were grown overnight in lysogeny broth (Luria-Bertani) [44] and the DNA was extracted using a Qiagen DNeasy Blood and Tissue kit (Qiagen; Hilden, Germany). Genomic libraries were prepared using an Illumina Nextera XT Library Preparation Kit according to the manufacturer instructions (Illumina; San Diego, USA). Sequencing was conducted using a Miseq v3 reagent kit on an Illumina Miseq®.

### Genome construction

Sequence read adaptors were trimmed with Cutadapt version 1.8.1 and quality trimmed using Trim Galore (q = 25 with ‘paired’ and ‘nextera’ flags) version 0.4.0 [45]. Contigs were assembled with SPAdes version 3.5.0 [46] (with kmers of 127, 117, 107, 97, 87, 77, 67 using the ‘careful’ flag), and annotated with Prokka version 1.11 [47] using the packaged database (using the ‘genus’ and ‘force’ flags). In addition to the 44 sequenced strains, sequence data for 6 Australian strains from DAR were processed with SPAdes and Prokka as above. Genome statistics including GC content, contig number, N50 and genome length were calculated with QUAST version 4.5 [48].

An additional 97 genomes of *Xanthomonas* strains available in GenBank were downloaded and re-annotated with Prokka (as described above) for standardisation and included in analyses (Table 1). These public genomes represent the majority of sequenced *X. arboricola*, *X. euvesicatoria* (*X. campestris* pv. *vesicatoria*), *X. gardneri*, *X. perforans*, and *X. vesicatoria* strains in GenBank. Average nucleotide identity of scaffolds was calculated for the entire dataset of 147 genomes with pyani version 0.2.4 [49] using the default settings. Strains were determined to belong to the same species if ANI values were above the 95–96% zone as set in Konstantinidis et al. [50] and utilised in Barak [29].

### Plasmid prediction and isolation

Plasmid prediction from the draft genomes of Australian strains was achieved using the plasmidSPAdes option (‘plasmid’ flag) of SPAdes version 3.8.0 [46]. Circular sequences from these assemblies were finished using recycler version 0.62 [51]. Bandage version 0.8.1 [52] was used to view the Recycler paths. The Blast+ algorithm version 2.6.0 [53] was used to compare the plasmid sequences to a custom database of complete *Xanthomonas* plasmids obtained from GenBank [54]. Plasmid isolation was carried out on a subset of strains (BRIP 38864, BRIP 62858, BRIP 62416, BRIP 62423, BRIP 62388, BRIP 62397, BRIP 63464, BRIP 38997) using the alkaline lysis method described in Chakrabarty [55]. The *Pseudomonas* strain DC 3000 [56] was used as an extraction control. Strains were grown in LB broth and processed with the described buffers, resuspending the pelleted DNA in distilled H20. Plasmid DNA was visualised on 0.7% agarose gels using standard electrophoresis at 40 V for 4–12 h.

### Analysis of genome content

A phylogeny that displays general species relationships was generated using the RedDog pipeline version V1beta.10.3 [57]. Briefly, the RedDog pipeline assembles and aligns raw reads against a reference genome (*X. campestris* pv. *vesicatoria* strain 85–10, Table 1), then creates a phylogeny using SNP data generated within the pipeline. Simulated reads were generated for public genomes using WgSim v. 0.3.1-r13 for inclusion in analysis [58]. Support values of the resulting phylogeny calculated by FastTree version 2.1.8 [59] are displayed as a range of 0 to 1. The final tree was annotated in FigTree ver. 1.4.2 [60] and GIMP version 2.8.14 [61].

Roary version 3.8.2 [62] was used to cluster homologues, generate a core alignment (−e and -n flags), and generate the pan-genus homologue matrix. The core alignment was then used to create individual phylogenies of BLS-causing species *X. euvesicatoria*, *X. perforans*, and *X. vesicatoria* with FastTree and annotated with FigTree and GIMP. The homologue matrix was then used to generate pan-genome pie plots using scripts available within the Roary package. Core genes were defined as present in 99–100% strains, soft core genes in 95–99% strains, shell genes in 15–95% strains and cloud genes in 0–15% strains. Gene discovery graphs were plotted using Roary scripts and R version 1.0.136 [62] to determine if the pan-genome was open or closed. These analyses were done for each species as defined in Table 1 (DAR 33341 was not included). The homologue matrix was filtered in R to identify unique genes of each species.

Carbohydrate-active and associated enzyme (CAZyme) coding sequences within each genome were identified using HMMER version 3.1b2 [63] and the DbCAN database [64]. The CAZyme hits were then clustered manually and presented as a heat map and dendrogram in R version 3.3.0 [65] using the pheatmap package [66] and annotated in GIMP. T3SS enzyme protein (effector) sequence and select regulatory protein sequence (rpf) were sourced from GenBank as listed in the *Xanthomonas* resource ‘effector’ page [67] and compared to the genome sequence and reconstructed plasmid sequence using the Blast+ algorithm. Hits were filtered (e-value 0.00001) and presented as a heat map using R and GIMP as described above.

## Additional files

Additional file 1: **Figure S1.** Phylogeny of Australian and Genbank genomes based on whole genome SNP data. Australian strains are indicated by BRIP and DAR collection prefixes; all others are public genomes of related species. Type strains are indicated in bold and branch support values are displayed to clade level (measured with the Shimodaira-Hasegawa test). Branch length is indicated by the scale bar. Clade colouring is based on phylogeny and ANI values to assign strains to species. The four Australian strains most closely related to *X. arboricola* are designated in the text as an uncharacterised *Xanthomonas* species. (PNG 278 kb)

Additional file 2: **Figure S2.** Gene discovery graphs for *X. euvesicatoria*, *X. perforans*, *X. vesicatoria* and *X. gardneri* plot number of new genes in the species pan-genome as genome number increases. The graph curves demonstrate how many new genes will be added with the addition of more sequenced genomes to estimate pan-genome completeness. X axis: genome number; Y axis: number of new genes. (PNG 45 kb)

Additional file 3: **Figure S3.** Standard electrophoresis of plasmid isolations with predicted circular sequence in base pairs below each lane. Ladder = Generuler™ DNA Ladder Mix, ThermoFisher Scientific, Waltham, Massachusetts. The 10 kbp label marks the largest ladder fragment, and the 60–70 kbp label marks the band present in DC 3000 (plasmid extraction control). A) gel was run for approx. 12 h at 40 V B) gel was run for approx. 4 h at 40 V. A and B represent two different extraction experiments (PNG 79 kb)

Additional file 4: **Table S1.** Homologues of effector protein families present in all strains of each *Xanthomonas* species and unique to each species. Effectors listed include all alleles displayed in the effector matrix for each effector family. ^a^ present in all strains of a species and possibly present in other strains/ species. ^b^ uncharacterised *Xanthomonas* sp. of four strains. ^c^ core to species in Schwartz et al. 2015. ^d^ core to species in Potnis et al. 2011. ^e^ Xp4B and Xp4p1S2 have truncated HpaG protein annotations, Xp4p1s2 has truncated XopAE protein annotation. ^f^ BRIP 39016 has a larger (650 aa) XopAE protein than all others (546 aa). ^g^ present in all Australian strains except BRIP 62397 (DOCX 13 kb)

Additional file 5: **Table S2.** Cazyme families present in all strains of each *Xanthomonas* species and families unique to each species. Function is described according to the CAZy database.^a^ all three present in DAR 26930, GH39 present in BRIP 39016, CE14 absent in GEV915. ^b^ genes of CAZyme family present in all strains of species. ^c^ genes of CAZyme family absent in all strains of species. ^d^ genes of CAZyme family present in some strains of species. ^e^ absent in 1–3 strains. (DOCX 13 kb)

## Abbreviations

AA: Auxiliary activity families
ANI: Average nucleotide identity
BLS: Bacterial leaf spot
BRIP: Queensland Plant Pathology Herbarium
CAZymes: Carbohydrate active enzymes
CBM: Carbohydrate binding modules
CE: Carbohydrate esterases
DAR: New South Wales Plant Pathology Herbarium
GH: Glycoside hydrolases
GT: Glycosyl transferases
NGS: Next generation sequencing
NSW: New South Wales
PL: Polysaccharide lyases
SNP: Single nucleotide polymorphism
T2SS: Type two secretion system
T3SS: Type three secretion system
TAL/ TALE: Transcription activator-like effectors
Xop: *Xanthomonas* outer protein
