# Supplementary figures and images for: Genomic sequence analysis reveals diversity of Australian Xanthomonas species associated with bacterial leaf spot of tomato, capsicum and chilli
Source: BMC Genomics. 2019 Apr 23;20:310. doi: 10.1186/s12864-019-5600-x (PMC6480910; doi:10.1186/s12864-019-5600-x)

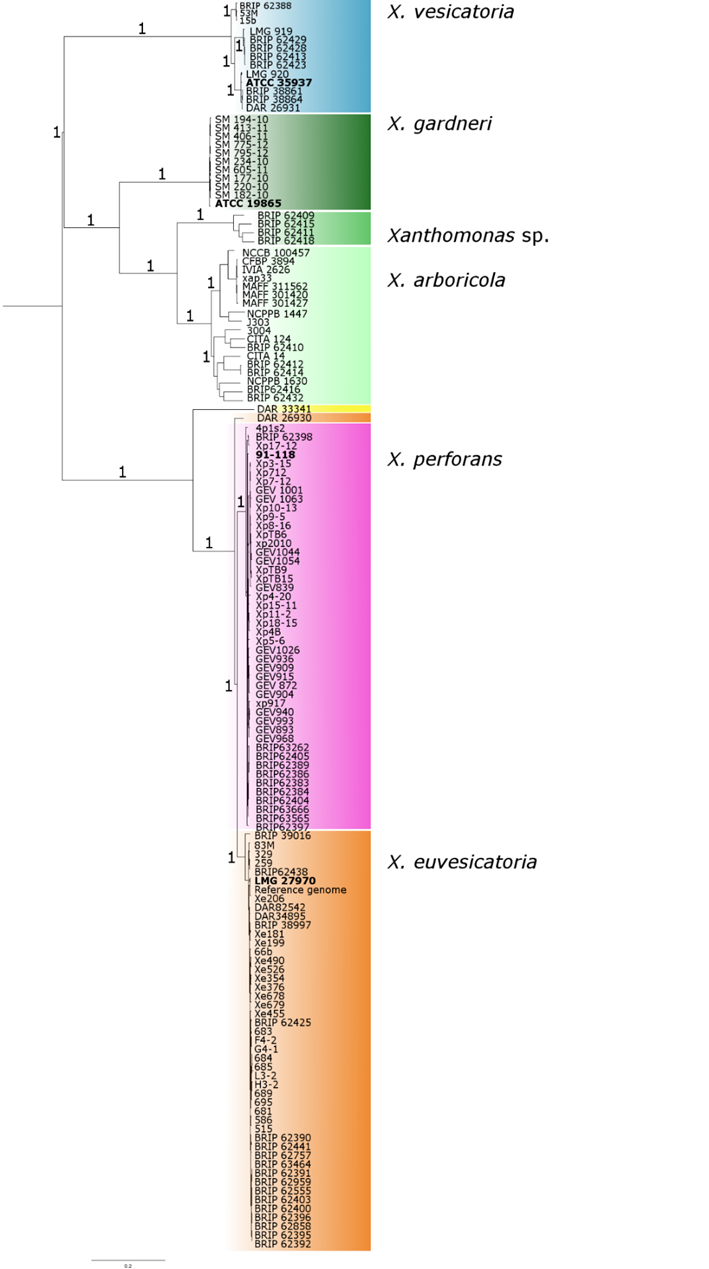

Supplement: Supplementary file 1 — Figure S1. Phylogeny of Australian and Genbank genomes based on whole genome SNP data. Australian strains are indicated by BRIP and DAR collection prefixes; all others are public genomes of related species. Type strains are indicated in bold and branch support values are displayed to clade level (measured with the Shimodaira-Hasegawa test). Branch length is indicated by the scale bar. Clade colouring is based on phylogeny and ANI values to assign strains to species. The four Australian strains most closely related to X. arboricola are designated in the text as an uncharacterised Xanthomonas species. (PNG 278 kb) [file 12864_2019_5600_MOESM1_ESM.png]

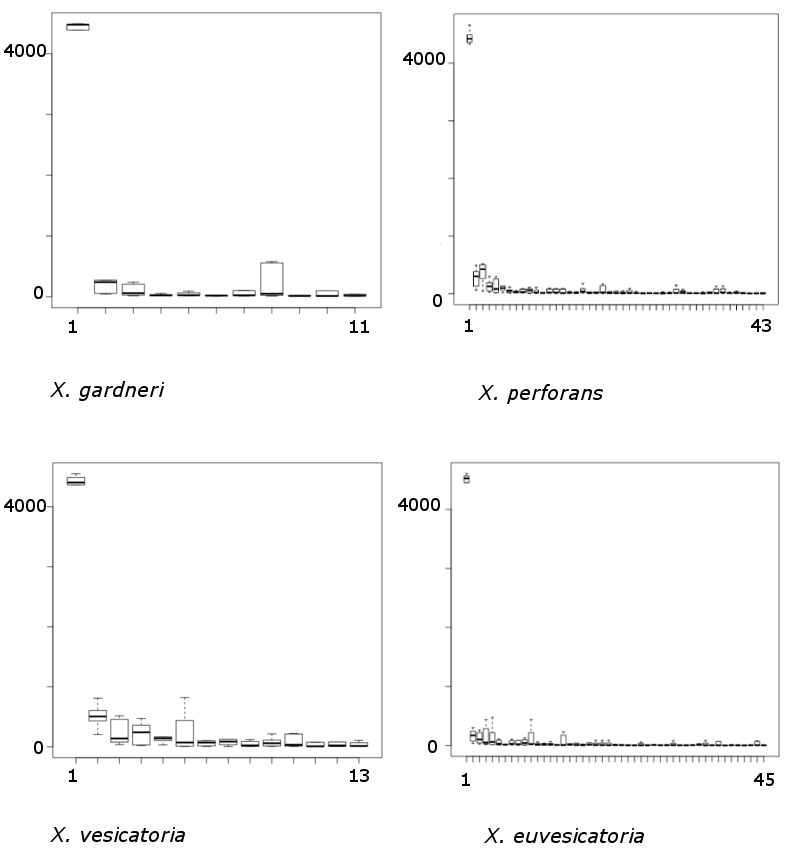

Supplement: Supplementary file 2 — Figure S2. Gene discovery graphs for X. euvesicatoria, X. perforans, X. vesicatoria and X. gardneri plot number of new genes in the species pan-genome as genome number increases. The graph curves demonstrate how many new genes will be added with the addition of more sequenced genomes to estimate pan-genome completeness. X axis: genome number; Y axis: number of new genes. (PNG 45 kb) [file 12864_2019_5600_MOESM2_ESM.png]

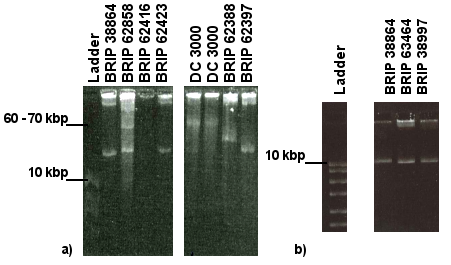

Supplement: Supplementary file 3 — Figure S3. Standard electrophoresis of plasmid isolations with predicted circular sequence in base pairs below each lane. Ladder = Generuler™ DNA Ladder Mix, ThermoFisher Scientific, Waltham, Massachusetts. The 10 kbp label marks the largest ladder fragment, and the 60–70 kbp label marks the band present in DC 3000 (plasmid extraction control). A) gel was run for approx. 12 h at 40 V B) gel was run for approx. 4 h at 40 V. A and B represent two different extraction experiments (PNG 79 kb) [file 12864_2019_5600_MOESM3_ESM.png]
