# Supplementary material for: Genomic sequence analysis reveals diversity of Australian Xanthomonas species associated with bacterial leaf spot of tomato, capsicum and chilli
Source: BMC Genomics. 2019 Apr 23;20:310. doi: 10.1186/s12864-019-5600-x (PMC6480910; doi:10.1186/s12864-019-5600-x)
Supplement: Supplementary file 4 — Table S1. Homologues of effector protein families present in all strains of each Xanthomonas species and unique to each species. Effectors listed include all alleles displayed in the effector matrix for each effector family. a present in all strains of a species and possibly present in other strains/ species. b uncharacterised Xanthomonas sp. of four strains. c core to species in Schwartz et al. 2015. d core to species in Potnis et al. 2011. e Xp4B and Xp4p1S2 have truncated HpaG protein annotations, Xp4p1s2 has truncated XopAE protein annotation. f BRIP 39016 has a larger (650 aa) XopAE protein than all others (546 aa). g present in all Australian strains except BRIP 62397 (DOCX 13 kb) [file 12864_2019_5600_MOESM4_ESM.docx]

|  | *X. euvesicatoria* | *X. perforans* | *X. vesicatoria* | *X. gardneri* | *X. arboricola* | *Xanthomonas sp.* ^b^ | All species |
| --- | --- | --- | --- | --- | --- | --- | --- |
| Present in all strains ^a^ | AvrBs2^cd^ | AvrBs2^cd^ | hrpW | hrpW | AvrBs2 | hrpW | AvrBs2 |
|  | HpaA | HpaA | xopG | xopG ^c^ | rpfF | xopA | rpfA |
|  | rpfA | rpfA | xopAI^d^ | xopAF | rpfA | AvrBs2 | rpfB |
|  | rpfB | rpfB | xopAG^d^ | AvrBs1 ^cd^ | rpfB | rpfF | rpfF |
|  | rpfC | rpfC | xopAL2 | AvrXccA1 |  | rpfA |  |
|  | rpfF | rpfF | rpfC | xopAM ^c^ |  | rpfB |  |
|  | xopA^c^ | xopA^c^ | xopAD^d^ | AvrXccA2 |  | xopF1 |  |
|  | xopAD^cd^ | xopAD^cd^ | AvrBs2^d^ | xopB ^c^ |  | hpaA |  |
|  | xopAE^c^ | xopAE^cd^ | rpfF | xopE3 |  | xopR |  |
|  | xopAJ | xopAK^c^ | rpfA | xopQ ^cd^ |  | xopZ1 |  |
|  | xopAK^c^ | xopC2^cd^ | rpfB | xopE2 ^c^ |  |  |  |
|  | xopC2 | xopE1^c^ | xopF1^d^ | xopD ^cd^ |  |  |  |
|  | xopE1^c^ | xopF1^cd^ | hpaA | rpfC |  |  |  |
|  | xopF1^cd^ | xopI^c^ | xopR | xopAD ^cd^ |  |  |  |
|  | xopI^c^ | xopL^cd^ | xopZ1^d^ | AvrBs2 ^cd^ |  |  |  |
|  | xopL^cd^ | xopN^cd^ | xopL^d^ | rpfF |  |  |  |
|  | xopN^cd^ | xopQ^cd^ | xopK^d^ | rpfA |  |  |  |
|  | xopP^c^ | xopR^cd^ | xopX^d^ | rpfB |  |  |  |
|  | xopQ^cd^ | xopV^c^ |  | hpaA |  |  |  |
|  | xopR^cd^ | xopX^cd^ |  | xopR ^cd^ |  |  |  |
|  | xopV^c^ | xopZ1^cd^ |  | xopZ1^d^ |  |  |  |
|  | xopX^cd^ | xopF2^c^ |  | xopL ^cd^ |  |  |  |
|  | xopZ1^cd^ | xopK^cd^ |  | xopK ^cd^ |  |  |  |
|  |  | xopD^cd^ |  | xopX ^cd^ |  |  |  |
|  | *X. euvesicatoria* | *X. perforans* | *X. vesicatoria* | *X. gardneri* | *X. arboricola* | *Xanthomonas sp.* ^b^ |  |
| XopAE | 36^f^ /48 | 44^e^/45 | 0/14 | 0/11 | 0/24 | 0/4 |  |
| HpaF | 12/48 | 0/45 | 0/14 | 0/11 | 0/24 | 0/4 |  |
| HpaG | 12/48 | 2e/45 | 0/14 | 0/11 | 0/24 | 0/4 |  |
| XopQ | 48/48 | 45/45 | 0/14 | 11/11 | 11/24 | 0/4 |  |
| AvrBsT | 2/48 | 37^g^/45 | 11/14 | 0/11 | 0/24 | 0/4 |  |
