# Supplementary material for: Genomic sequence analysis reveals diversity of Australian Xanthomonas species associated with bacterial leaf spot of tomato, capsicum and chilli
Source: BMC Genomics. 2019 Apr 23;20:310. doi: 10.1186/s12864-019-5600-x (PMC6480910; doi:10.1186/s12864-019-5600-x)
Supplement: Supplementary file 5 — Table S2. Cazyme families present in all strains of each Xanthomonas species and families unique to each species. Function is described according to the CAZy database.a all three present in DAR 26930, GH39 present in BRIP 39016, CE14 absent in GEV915. b genes of CAZyme family present in all strains of species. c genes of CAZyme family absent in all strains of species. d genes of CAZyme family present in some strains of species. e absent in 1–3 strains. (DOCX 13 kb) [file 12864_2019_5600_MOESM5_ESM.docx]

| **Group absent in *X. euvesicatoria* and *X. perforans*** | | | | **function** | | | |
| --- | --- | --- | --- | --- | --- | --- | --- |
| GH6 | | | | Bacterial Endoglucanase cellobiohydrolase | | | |
| GH104 | | | | lytic transglyosylases | | | |
| AA12 | | | | oxidoreductase | | | |
| GT27 | | | | polypeptide α-N-acetylgalactosaminyltransferase | | | |
| PL10 | | | | pectate lyase | | | |
| PL3 | | | | pectate lyase | | | |
| CBM63 | | | | May bind cellulose | | | |
| GH16 | | | | Acts on glycosidic bonds | | | |
| PL17 | | | | alginate lyase etc | | | |
| PL6 | | | | alginate lyase etc | | | |
| GH4 | | | | Bacterial glycosidases | | | |
| GH89 | | | | α-N-acetylglucosaminidase | | | |
| GH84 | | | | β-N-acetylglucosaminidases etc | | | |
| **Absent in *X. euvesicatoria* and present in *X. perforans* strains ^a^** | | | | | | | |
| CE8 | | | | pectin methylesterase | | | |
| CE14 | | | | Deacetylase etc | | | |
| GH39 | | | | Cleave glycosidic bonds; β-xylosidase and α-L-iduronidase | | | |
| **Unique to *X. vesicatoria* and DAR 33341** | | | | | | | |
| GH6 | | | | Bacterial Endoglucanase cellobiohydrolase | | | |
| **CAZyme families from Potnis et al. 2011 – detection in this study** | | | | | | | |
|  | ***X. euvesicatoria*** | ***X. perforans*** | ***X. vesicatoria*** | | ***X. gardneri*** | ***X. arboricola*** | ***Xanthomonas sp.*** |
| GH10 | + ^b^ | + | + | | + | + | + |
| GH67 | + | + ^*^ | + | | + | + | + |
| GH51 | + | + | + | | + | + | + |
| GH5 | + | + | + | | + | + | + |
| GH8 | + ^*^ | + | + ^*^ | | -^c^ | ~ ^d^ | - |
| GH9 | + ^*^ | + | + | | + | + | + |
| PL1 | + | + | + | | + | + | + |
| PL3 | - | - | + | | + | ~ | + |
| PL4 | + | + | + | | + | + ^e^ | - |
| PL9 | + | + ^*^ | + | | + | - | - |
| PL10 | - | - | + ^*^ | | + | ~ | - |
